# Land use drives trematode dynamics in a restored stream system

**DOI:** 10.1016/j.crpvbd.2026.100357

**Published:** 2026-02-13

**Authors:** Annabell Hüsken, Jessica Schwelm, Bernd Sures

**Affiliations:** a Aquatic Ecology and Centre for Water and Environmental Research, University of Duisburg-Essen, Universitätsstraße 5, Essen, 45141, Germany; b Research Center One Health Ruhr, Research Alliance Ruhr, University of Duisburg-Essen, Universitätsstraße 5, Essen, 45141, Germany

**Keywords:** Trematodes, Freshwater, Degradation, Restoration, Land use, Urban, *Ampullaceana balthica*

## Abstract

Urban freshwater ecosystems are subjected to multiple anthropogenic and natural stressors, emphasizing the need to evaluate and restore their ecological status amid ongoing biodiversity loss. While successional dynamics of free-living taxa are commonly assessed, the environmental drivers shaping parasite-host interactions in degraded and restored systems remain poorly understood. Here, we examined trematode species richness, overall prevalence, and community composition in first intermediate snail hosts across an urban stream system with a history of severe degradation and gradual restoration. Over two years of monthly sampling, we assessed differences in trematode dynamics across restoration stages and adjacent land-use types. We examined 6300 snails of 13 different species and identified 25 trematode species with an overall prevalence of 19.1%. Adjacent land use emerged as a consistent predictor of trematode dynamics, with agricultural sites supporting higher species richness and prevalence than forested or urban sites. In contrast, trematode community composition showed limited spatial turnover and did not differ substantially with restoration history or land use. Restoration history was not associated with trematode richness, overall prevalence, or community composition, suggesting quick recovery of trematode-host assemblages in recently restored habitats. The most abundant snail, *Ampullaceana balthica*, was identified as a key host species, harboring the majority of trematode taxa and the highest infection prevalence. Overall, our results highlight land use as a dominant landscape-level driver of trematode dynamics in urban freshwater ecosystems and support the potential of trematodes as complementary bioindicators for assessing ecological conditions in restored habitats.

## Highlights

• Two-year study of snail-trematode dynamics in a restored urban freshwater system.
• Agricultural land use increases trematode richness and prevalence.
• Trematode communities establish rapidly, independent of restoration history.
• *Ampullaceana balthica* as a key host with the highest trematode diversity and prevalence.

## Graphical abstract

**Figure:**
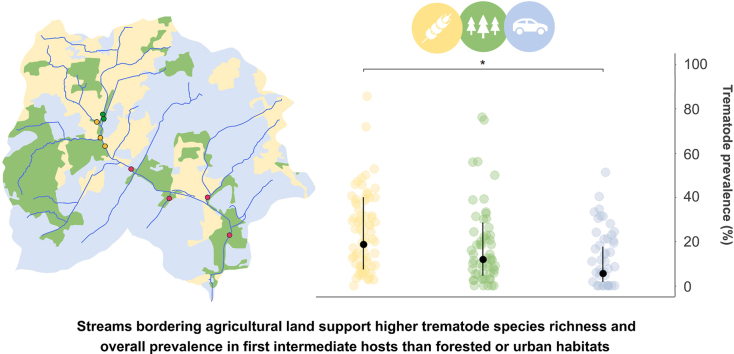

## 1 Introduction

Freshwater ecosystems are among the most threatened habitats globally, facing severe biodiversity loss driven by a combination of natural and anthropogenic stressors (Birk et al., 2020; Sures et al., 2023). Urban stream ecosystems are particularly vulnerable, as they are subjected to multiple stressors including elevated nutrient loads, pollution, hydromorphological modification, and land-use change associated with high human population densities (Dudgeon, 2019; Reid et al., 2019; Schürings et al., 2022; Kaijser et al., 2025). Consequently, assessing and restoring the ecological status of urban freshwaters has become an environmental priority (Haase et al., 2023). Biological monitoring typically relies on free-living diversity, such as benthic invertebrates and fish, which serve as well-established indicators of degradation and recovery (Violin et al., 2011; Pander and Geist, 2013; Gillmann et al., 2024). Complementary to free-living organisms, parasites have increasingly been recognized as indicators of ecosystem integrity, as they are able to reflect ecosystem processes across multiple host taxa and trophic interactions (Hechinger et al., 2007; Moore et al., 2024). Nonetheless, the relative importance of environmental drivers structuring parasite-host dynamics remains insufficiently understood, limiting the application of parasites as indicators of freshwater degradation and recovery (Prati et al., 2022; Sures et al., 2025a).

Digenean trematodes are among the most diverse and widespread metazoan parasites (Esch et al., 2002). Their heteroxenous life cycles are typically water-bound, as aquatic molluscs play a key role as the primary first intermediate host. A broad range of metazoans may serve as second intermediate hosts, while the definitive host of trematodes is a vertebrate (Huspeni and Lafferty, 2004; Sures et al., 2025b). Successful transmission, therefore, requires sequential availability of multiple hosts, and trematodes in first intermediate hosts have already been implemented as promising cross-taxon indicators of habitat quality and free-living diversity across aquatic ecosystems (e.g. Huspeni and Lafferty, 2004; Hechinger et al., 2007; Santiago Bass and Weis, 2008; Aguirre-Macedo et al., 2011; Gordy et al., 2020; Zemmer et al., 2020; Schwelm et al., 2021; Moore et al., 2023; Moore et al., 2024; Hüsken et al., 2025). Following restoration of degraded habitats, trematode diversity and prevalence are expected to increase, as host communities recover and habitats become more attractive to intermediate and definitive hosts (Huspeni and Lafferty, 2004; Santiago Bass and Weis, 2008; Moore et al., 2023). However, trematode recovery can be delayed or incomplete, resulting in decreased species richness and prevalence even years after the restoration (Morley and Lewis, 2006). As with diversity of free-living species, such patterns may indicate incomplete recovery of host networks and trophic interactions, or environmental stressors persisting after restoration measures. Urban streams, in particular, often experience high levels of anthropogenic alteration and have been associated with reduced trematode prevalence and species richness in snail hosts (Altman and Byers, 2014). Thus, trematode responses to restoration in urban settings may depend on the specific characteristics of restored habitats (e.g. Morley and Lewis, 2006). For instance, nutrient enrichment and eutrophication may increase snail abundance and promote trematode transmission, and agricultural land use has been associated with a shift in trematode assemblages towards livestock parasites (Johnson et al., 2007; Altman and Byers, 2014; Hammoud et al., 2025). However, disentangling the effects of these environmental drivers to get a clear picture on trematode restoration dynamics is further complicated by additional factors influencing trematode dynamics, as infection in first intermediate hosts may vary across seasons (e.g. Zemmer et al., 2017), snail host diversity and abundance (e.g. Gordy et al., 2020; McPhail et al., 2025), or snail host size (e.g. Graham, 2003; Tolley-Jordan and Chadwick, 2019).

To assess trematodes as indicators of restoration and freshwater ecosystem health in anthropogenic landscapes, we investigated trematode dynamics in first intermediate snail hosts across an urban stream system with a history of severe degradation and gradual restoration. By integrating restoration history and land-use characteristics with additional environmental drivers known to influence trematode dynamics, we quantified how trematode species richness, overall prevalence, and community composition vary across the catchment. Specifically, we tested whether (H1) trematode species richness and overall prevalence increase with time since restoration; (H2) trematode species richness and overall prevalence are lower at highly urbanized sites; and (H3) trematode community composition varies among sites with different restoration histories and land-use types, potentially reflecting differences in habitat use by definitive hosts.

## 2 Materials and methods

### 2.1 Study site

**Fig. 1:**
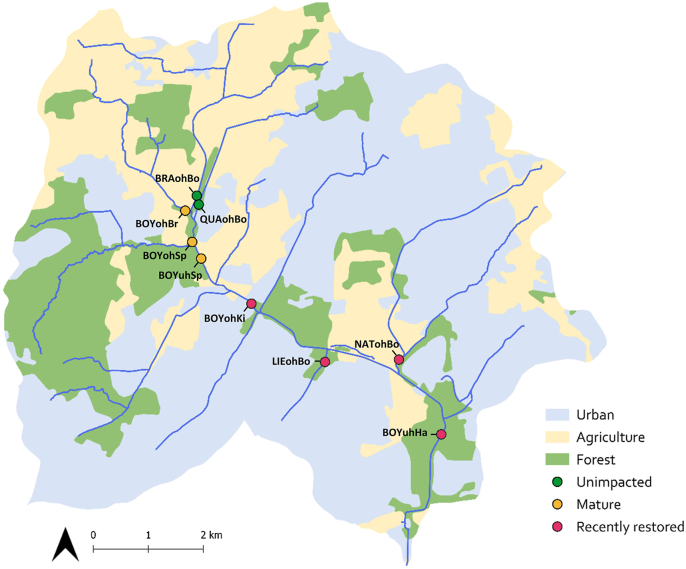
Map of the Boye catchment, with the three major types of land use (based on CLC data) in the catchment given as urban (*blue*), agriculture (*yellow*) and forest (*green*). Sampling sites are indicated by colored dots, representing the restoration history of sites (unimpacted, mature, recently restored). Map generated in QGIS version 3.42.0-Münster.

The Boye catchment is located in the Ruhr Metropolitan Area in North Rhine-Westphalia, Germany, and drains into the River Emscher, which subsequently flows into the River Rhine. With a total stream length of 90 km, it drains an area of 77 km^2^. Upstream sections of the Boye catchment are surrounded by agricultural fields and urban forest, while the downstream sections pass through highly urbanized areas (Fig. 1) (Gillmann et al., 2024). As industrial activity in the Ruhr region intensified in the early 20th century, the Boye stream and its main tributaries underwent severe anthropogenic alteration and were converted into concrete open sewer channels over a stretch of 30.5 km to handle domestic and industrial wastewater. Beginning in 1993, a large-scale restoration project was initiated, which included redirection of wastewater into underground channels, allowing for the gradual removal of the concrete riverbed and subsequent restoration of the channels and riparian areas (Winking et al., 2014, 2016). By the end of 2021, the Boye catchment was free of wastewater input and hydromorphological restoration of all sections was finalized in 2024. The former sewer channels were recolonized by benthic invertebrate communities from unaffected sites following the implementation of restoration measures (Winking et al., 2016; Gillmann et al., 2023, 2024, 2025).

Within the framework of the Collaborative Research Centre RESIST (https://sfb-resist.de), we selected nine sampling sites in the catchment for the investigation of trematode communities based on the stream’s restoration history, adjacent land use, and the presence of abundant snail populations. In line with Gillmann et al. (2025), the sites were categorized into three groups: unimpacted (sites that never carried wastewater); mature (sites that were restored more than 10 years prior to sampling); and recently restored (sites that were restored less than 5 years prior to sampling) (Table 1, Fig. 1). Land use in proximity to the sites was assessed using Corine Land Cover (CLC) 2018 data, grouped into the three main categories (urban, agriculture, and forest). Land-use classifications were validated and, if necessary, adjusted based on on-site observations (Table 1). To assess water quality at each site, we further measured the following water parameters at all sampling events: pH; temperature (°C); conductivity (μS/cm); and oxygen (mg/l) (Supplementary file 1).

**Table 1:** Sampling sites in the Boye catchment with the indication of wastewater removal and hydromorphological restoration history, adjacent land use, and site group classification (restoration history).

Stream name	Site ID	Coordinates	No. of snails	Wastewater-free	Hydromorphological restoration	Land use	Site group
Brabecker Mühlenbach	BRAohBo	51.571194°N, 6.930556°E	964	–	–	Agriculture	Unimpacted
Quälingsbach	QUAohBo	51.569667°N, 6.931111°E	379	–	–	Forest	
Boye	BOYohBr	51.569135°N, 6.927698°E	160	2007	2002	Forest	Mature
Boye	BOYohSp	51.564297°N, 6.930277°E	718	2007	2002	Forest	
Boye	BOYuhSp	51.561336°N, 6.932617°E	865	2007	2002	Agriculture	
Boye	BOYohKi	51.554079°N, 6.946715°E	460	2017	2021	Urban	Recently restored
Liesenfeldbach	LIEohBo	51.546008°N, 6.965355°E	983	2017	2021	Agriculture	
Nattbach	NATohBo	51.546348°N, 6.98579°E	951	2017	2021	Forest	
Boye	BOYuhHa	51.534515°N, 6.99774°E	820	2017	2023	Urban	

### 2.2 Sampling

Between April 2023 and July 2025, we collected and screened 6300 individual snails for trematode infections during year-round monthly samplings in the Boye catchment. Snails were hand-picked from sediments, stones, and deadwood along the riverbed and shoreline or collected with hand nets from submersed vegetation. To ensure standardization, equal sampling effort defined as people and time spent sampling was maintained across all sites and for all snail species. Collected snails were transported to the laboratory, measured and individually placed in beakers filled with filtered water (500 μm mesh size) separately collected from the study catchment. The snails were kept at room temperature under continuous light exposure for three consecutive days to stimulate cercarial emergence from infected individuals. Each beaker was examined daily under a stereomicroscope for the presence of patent infections. Snails collected between May 2023 and July 2024 were marked with water-based enamel paint (Wong et al., 2013) and returned to their respective habitats after the three-day period as part of a mark-release-recapture (MRR) study in the catchment. Recapture events were not included in the present dataset. Snails collected between August 2024 and July 2025 were dissected for detection of prepatent infections with sporocysts or rediae. Consequently, our dataset comprises one full year of patent infections only and one full year including both patent and prepatent infections, which was considered during statistical analyses.

### 2.3 Species identification and phylogenetic analyses

Snail species were morphologically identified using the taxonomic key by Glöer (2017), and representative specimens of infected species were validated molecularly (15 isolates; accession numbers PX916090-PX916093 and PX919747-PX919757). Preliminary morphological identification of trematode species was conducted on live cercariae under a light microscope (Olympus BX51, Tokyo, Japan) using trematode larval identification keys and other relevant literature (Faltýnková et al., 2007, 2008; Schwelm et al., 2020; Hüsken et al., 2025). Photomicrographs of live cercariae were taken with a digital camera (Olympus UC30, Tokyo, Japan) attached to the microscope. All trematode samples were preserved in 96% ethanol for molecular identification.

Total genomic DNA was extracted from the foot tissue of selected snails and pooled trematode cercarial samples using a salt precipitation protocol (Grabner et al., 2015) as described by Schwelm et al. (2020). Molecular identification of snail species was conducted on the cytochrome *c* oxidase subunit 1 (*cox*1) Folmer barcoding region or a section of the 28S rRNA gene (Supplementary file 2: Table S2). Molecular identification of trematodes was conducted on the D1**-**D3 domains of the nuclear 28S rRNA gene and/or a section of the mitochondrial *cox*1 or NADH dehydrogenase subunit 1 (*nad*1) gene (Supplementary file 2: Table S2). All PCR reactions were carried out in a 20-μl reaction volume with 10 μl DreamTaq™ Hot Start Green PCR Master Mix, 1.6 μl of each primer (10 μM), 4.8 μl molecular-grade water, and 2 μl DNA (approximately 1.5 ng/μl). Thermocycling conditions followed the protocols described in the source papers (Supplementary file 2: Table S2). PCR products were purified and sequenced at Microsynth Seqlab (Germany) using the respective forward primer (20 μM). Sequence data were quality-checked, edited and aligned using the MUSCLE algorithm (Edgar, 2004) implemented in Geneious Prime v.2024.0.5 (https://www.geneious.com) and subsequently compared against the NCBI database using the Basic Local Alignment Search Tool (BLAST) (www.ncbi.nih.gov/BLAST/) to assign a preliminary species ID. Three to five selected isolates of each distinct snail and trematode species (> 95% high-quality base calls) were selected for bidirectional Sanger sequencing using the PCR primers and additional sequencing primers (20 μM) (Supplementary file 2: Table S2), and edited and assembled in Geneious Prime. Where possible, these isolates were selected to represent each snail-trematode species combination. However, bidirectional sequencing was unsuccessful for three combinations (*Echinoparyphium recurvatum* in *Lymnaea stagnalis*, *Australapatemon burti* in *L. stagnalis*. *A. burti* in *Stagnicola palustris*). The newly generated trematode sequences (124 isolates) were deposited as molecular vouchers in the NCBI database under the accession numbers PX641028-PX641093 (28S), PX637199-PX637240 (*cox*1), and PX648493-PX648508 (*nad*1).

Based on cercarial morphology, the results of BLAST analyses of the new sequences and the availability of suitable reference data in GenBank, eight molecular datasets for 28S rDNA and three molecular datasets for mitochondrial markers were generated for phylogenetic analyses. Alignments were conducted with the newly generated molecular vouchers as well as published sequences from GenBank using the MUSCLE algorithm (Edgar, 2004) implemented in Geneious Prime. Mitochondrial sequences were screened for pseudogene amplification using the trematode mitochondrial code (translation table 21; https://www.ncbi.nlm.nih.gov/taxonomy/utils/wprintgc.cgi#SG21) (Garey and Wolstenholme, 1989; Ohama et al., 1990). All alignments were trimmed to the shortest sequence length, and uncorrected p-distances calculated using MEGA 11 (Tamura et al., 2021) (Supplementary file 3). Phylogenetic trees for each dataset were constructed using Maximum Likelihood (ML) and Bayesian Inference (BI) analyses. For the ML analyses, the best-fitting nucleotide substitution models were identified using the Akaike information criterion (AIC) in ModelFinder (Kalyaanamoorthy et al., 2017) (Supplementary file 2: Table S3). ML analyses were performed using IQ-TREE v.2.4.0 (Minh et al., 2020) with 1000 bootstrap pseudoreplicates. For the BI analyses, the best-fitting nucleotide substitution models were identified using the AIC in MrModelTest v.2.4 (Nylander, 2004) (Supplementary file 2: Table S3). BI analyses were performed using MrBayes v.2.3.7 (Ronquist et al., 2012), sampling Markov chain Monte Carlo chains every 1000 generations for 10,000,000 generations and retaining only the final 75% of trees for the consensus. All trees were visualized using FigTree v.1.4.4 (http://tree.bio.ed.ac.uk/software/figtree/) and are presented in Supplementary file 2.

### 2.4 Data analyses

Statistical analyses were performed in RStudio v.2024.12.0.467 (Posit Team, 2024) based on R v.4.4.2 (R Core Team, 2024) with significance assumed at *P* < 0.05. First, we examined the trematode fauna associated with each snail host species and individual sampling site. Trematode species richness was calculated as the total number of trematode species detected across all examined host individuals. Trematode prevalence was calculated as the number of hosts infected with a certain trematode species, relative to the total number of hosts examined (Bush et al., 1997). Overall trematode prevalence per site was calculated as the proportion of all hosts infected, regardless of trematode species.

To test whether restoration history and adjacent land use influenced trematode species richness and overall prevalence (H1, H2), we fitted generalized linear mixed-effects models (GLMMs) using the *glmmTMB* package v.1.1.11 (Brooks et al., 2017) with trematode species richness and overall trematode prevalence as the respective response variables. First, models were fitted using data from all snail host species, with fixed effects including site group (restoration history) and land use as well as season, snail species richness, and water parameters. For the factor “season”, samples were grouped into spring (March-May), summer (June-August), autumn (September-November), and winter (December-February). All models further included the factor “method” to account for different approaches to trematode detection (MRR *vs* dissection). We then fitted additional models using only *Ampullaceana balthica*, the most abundant host species present at all sites. These models included the same fixed effects as above, with the addition of mean snail size as an indicator of age, commonly correlated with infection (e.g. Graham, 2003; Tolley-Jordan and Chadwick, 2019). In all models, trematode species richness was modeled using a negative binomial distribution (log link) and overall prevalence using a binomial error distribution (logit link). To account for repeated monthly sampling, we included nested random intercepts for month/site and year/site. Model fit and assumptions were assessed using *DHARMa* v.0.4.7 (Hartig, 2024) and explained variance (R^2^c) was calculated using *MuMIn* v.1.48.11 (Bartoń, 2025). Fixed effects and uncertainty estimates were visualized using the *parameters* package v.0.24.2 (Lüdecke et al., 2020). For factors with ≥ 2 levels (land use, site group, season), we computed estimated marginal means (EMMs) using *emmeans* v.1.11.0 (Lenth, 2025) and conducted pairwise comparisons using Tukey's honestly significant difference (HSD).

To test whether these patterns are reflected across trematode component communities (H3), we analyzed trematode dynamics at the component community level, defined as all trematode species infecting a given host species at a given time and place (Bush et al., 1997). Shannon diversity was calculated using *vegan* v.2.6.10 (Oksanen et al., 2025) and modeled using a GLMM with a Tweedie error distribution (log link). Fixed effects included site group, land use, water parameters, season, method, host species, and mean host size. Subsequently, we fitted a multivariate abundance model using a generalized latent variable mixed model (GLVMM) in *glmmTMB*, following McGillycuddy et al. (2025). Trematode abundance (number of infected hosts per trematode species) was modeled using a negative binomial distribution and the same fixed effects as above. Prior to including fixed effects, we derived an unconstrained ordination of trematode component communities from a reduced-rank latent variable random effect to estimate two-dimensional covariance in trematode abundance across component communities. Both models included nested random intercepts for month/site and year/site and were evaluated using *DHARMa* and *MuMIn* packages. Group-level differences were assessed as described above.

## 3 Results

### 3.1 Occurrence of snails and trematodes

**Fig. 2:**
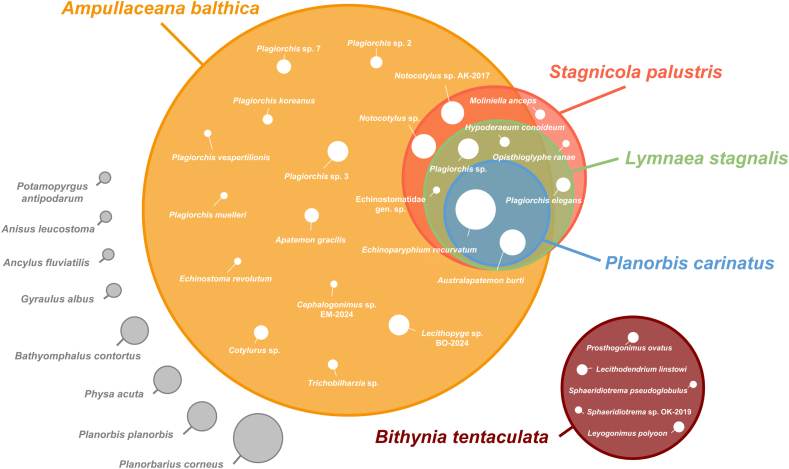
Visual representation of snail species and their associated trematode species in the Boye catchment. Each colored circle corresponds to a snail species, with its area proportional to the total number of individuals sampled. White circles within each colored circle represent trematode species detected in that host, with circle size scaled to the frequency of infections. Overlapping areas between circles indicate trematode species shared among host species.

In our survey of 6300 snails, we identified 13 snail species from five families. Planorbid snails were represented by six species, *Anisus leucostoma* (*n* = 2), *Bathyomphalus contortus* (*n* = 25), *Gyraulus albus* (*n* = 4), *Planorbis carinatus* (*n* = 302), *Planorbis planorbis* (*n* = 56), and *Planorbarius corneus* (*n* = 85). Lymnaeid snails were represented by three species, *Ampullaceana balthica* (*n* = 4058), *Lymnaea stagnalis* (*n* = 433) and *Stagnicola palustris* (*n* = 803). The other families were represented by one species each, *Ancylus fluviatilis* (Ancylidae, *n* = 2), *Bithynia tentaculata* (Bithyniidae, *n* = 488), *Physa acuta* (Physidae, *n* = 43), and *Potamopyrgus antipodarum* (Tateidae, *n* = 1) (Fig. 2, Table 2).

**Table 2:** Prevalence of the recorded trematode species in the respective snail host species summarized from all samplings between 2023 and 2025. Life cycle reconstructions for the second intermediate and definitive host groups are based on literature data.

Trematode family and species	Snail species	Prevalence in % (*n*)	GenBank accession number		Second intermediate host group	Definitive host group	Reference
			28S rDNA	*cox*1/*nad*1^a^			
**Family Cephalogonimidae**							
*Cephalogonimus* sp. EM-2024 *sensu*Hüsken et al. (2025)	*A. balthica*	0.07 (3)	PX641077; PX641080	PX637233; PX637235	Amphibians	Amphibians	Lang (1968); Dronen and Lang (1974); Tepe and Yilan (2021)
**Family Echinostomatidae**							
*Echinoparyphium recurvatum*	*A. balthica*	10.4 (422)	PX641028; PX641060; PX641073	PX648493^a^; PX648501^a^; PX648504^a^	Molluscs	Waterfowl	Brown et al. (2011); Selbach et al. (2020)
	*L. stagnalis*	0.7 (3)	–	–			
	*S. palustris*	0.6 (5)	PX641046; PX641047	PX648497^a^; PX648498^a^			
	*P. carinatus*	0.3 (1)	–	PX648502^a^			
*Echinostoma revolutum*	*A. balthica*	0.02 (1)	PX641037	PX648495^a^	Molluscs, amphibians	Waterfowl	Brown et al. (2011); Selbach et al. (2020)
Echinostomatidae gen. sp.	*A. balthica*	0.02 (1)	PX641091	PX648506^a^	Unknown	Unknown	–
	*L. stagnalis*	0.2 (1)	PX641093	PX648508^a^			
	*S. palustris*	0.1 (1)	PX641092	PX648507^a^			
*Hypoderaeum conoideum*	*A. balthica*	0.07 (3)	PX641054	PX648499^a^; PX648500^a^	Molluscs	Waterfowl	Brown et al. (2011)
	*L. stagnalis*	0.2 (1)	PX641035	PX648494^a^			
	*S. palustris*	0.1 (1)	–	PX648505^a^			
*Moliniella anceps*	*S. palustris*	0.2 (2)	PX641038; PX641072	PX648496^a^; PX648503^a^	Molluscs	Rallid birds	Brown et al. (2011)
**Family Lecithodendriidae**							
*Lecithodendrium linstowi*	*B. tentaculata*	2.7 (13)	PX641056; PX641078; PX641082	PX637218; PX637234; PX637236	Insect larvae	Bats	Enabulele et al. (2018); Lotz (2024)
**Family Notocotylidae**							
*Notocotylus* sp. (not further identified)	*A. balthica*	2.4 (97)	–	–	None (encyst on vegetation/substrate)	Waterfowl	Brown et al. (2011); Osnas and Lively (2011)
	*S. palustris*	0.2 (2)	–	–			
*Notocotylus* sp. AK-2017 *sensu*Soldánová et al. (2017)	*A. balthica*	3.8 (155)	PX641062; PX641090	–	None (encyst on vegetation/substrate)	Waterfowl	Brown et al. (2011); Osnas and Lively (2011); Soldánová et al. (2017)
	*S. palustris*	0.9 (7)	PX641066; PX641067; PX641069	PX637225			
**Family Plagiorchiidae**							
*Lecithopyge* sp. BO-2024 *sensu*Hüsken et al. (2025)	*A. balthica*	1.3 (53)	PX641053; PX641071; PX641074	PX637217; PX637229; PX637230	Amphibians	Amphibians	Grabda-Kazukska (1969)
*Plagiorchis* sp. (not further identified)	*A. balthica*	1.3 (53)	–	–	–	–	–
	*L. stagnalis*	2.3 (10)	–	–			
	*S. palustris*	0.6 (5)	–	–			
*Plagiorchis* sp. 2 *sensu*Soldánová et al. (2017)	*A. balthica*	0.2 (10)	PX641049; PX641051; PX641065	PX637213; PX637215; PX637224	Molluscs, crustaceans	Unknown	Soldánová et al. (2017)
*Plagiorchis* sp. 3 *sensu*Soldánová et al. (2017)	*A. balthica*	1.8 (72)	PX641041; PX641043; PX641044	PX637207; PX637209; PX637210	Insect larvae	Unknown	Soldánová et al. (2017)
*Plagiorchis* sp. 7 *sensu*Soldánová et al. (2017)	*A. balthica*	1.2 (49)	PX641050; PX641083; PX641089	PX637214; PX637237; PX637240	Unknown	Unknown	Soldánová et al. (2017)
*Plagiorchis elegans*	*L. stagnalis*	2.5 (11)	PX641033; PX641036; PX641081	PX637202; PX637204	Aquatic arthropods	Birds, mammals, reptiles	Brown et al. (2011); Selbach et al. (2020)
	*S. palustris*	1.0 (8)	PX641052	PX637216			
*Plagiorchis koreanus*	*A. balthica*	0.05 (2)	PX641040; PX641042	PX637206; PX637208	Insect larvae	Bats	Kirillova et al. (2024)
*Plagiorchis muelleri*	*A. balthica*	0.02 (1)	PX641061	PX637221	Insect larvae	Bats	Kirillova et al. (2024)
*Plagiorchis vespertilionis*	*A. balthica*	0.02 (1)	PX641068	PX637226	Insect larvae	Bats	Kirillova et al. (2024)
**Family Pleurogenidae**							
*Leyogonimus polyoon*	*B. tentaculata*	1.0 (5)	PX641030; PX641031; PX641034	PX637200; PX637201; PX637203	Molluscs	Rallid birds	Roy and St-Louis (2017); Schwelm et al. (2020)
**Family Prosthogonimidae**							
*Prosthogonimus ovatus*	*B. tentaculata*	0.6 (3)	PX641088	PX637239	Insect larvae	Birds	Heneberg et al. (2015)
**Family Psilostomidae**							
*Sphaeridiotrema pseudoglobulus*	*B. tentaculata*	0.6 (3)	PX641076; PX641087	PX637232; PX637238	Molluscs	Waterfowl	Sandland et al. (2014)
*Sphaeridiotrema* sp. OK-2019 *sensu*Schwelm et al. (2020)	*B. tentaculata*	1.2 (6)	PX641084; PX641085; PX641086	–	Molluscs	Waterfowl	Schwelm et al. (2020)
Schistosomatidae							
*Trichobilharzia* sp.	*A. balthica*	0.2 (8)	PX641039; PX641045; PX641059	PX637205; PX637211; PX637220	None (direct infection)	Waterfowl	Brown et al. (2011), Selbach et al. (2020)
**Family Strigeidae**							
*Apatemon gracilis*	*A. balthica*	0.8 (33)	PX641029; PX641032; PX641048	PX637199; PX637212	Fishes	Fish-eating birds	Selbach et al. (2020)
*Australapatemon burti*	*A. balthica*	3.5 (143)	PX641070; PX641075; PX641079	PX637227; PX637231	Leeches	Waterfowl	Brown et al. (2011)
	*L. stagnalis*	0.2 (1)	–	–			
	*S. palustris*	0.1 (1)	–	–			
	*P. carinatus*	2.3 (7)	–	PX637228			
*Cotylurus* sp.	*A. balthica*	0.7 (30)	PX641057; PX641058; PX641063; PX641064	PX637219; PX637222; PX637223	Leeches, molluscs	Waterfowl	Pyrka et al. (2022)
**Family Telorchiidae**							
*Opisthioglyphe ranae*	*S. palustris*	0.1 (1)	PX641055	–	Amphibians, molluscs	Amphibians	Brown et al. (2011)

*Abbreviation*: *n*, number of infected snails.

a *nad*1 sequences.

**Fig. 3:**
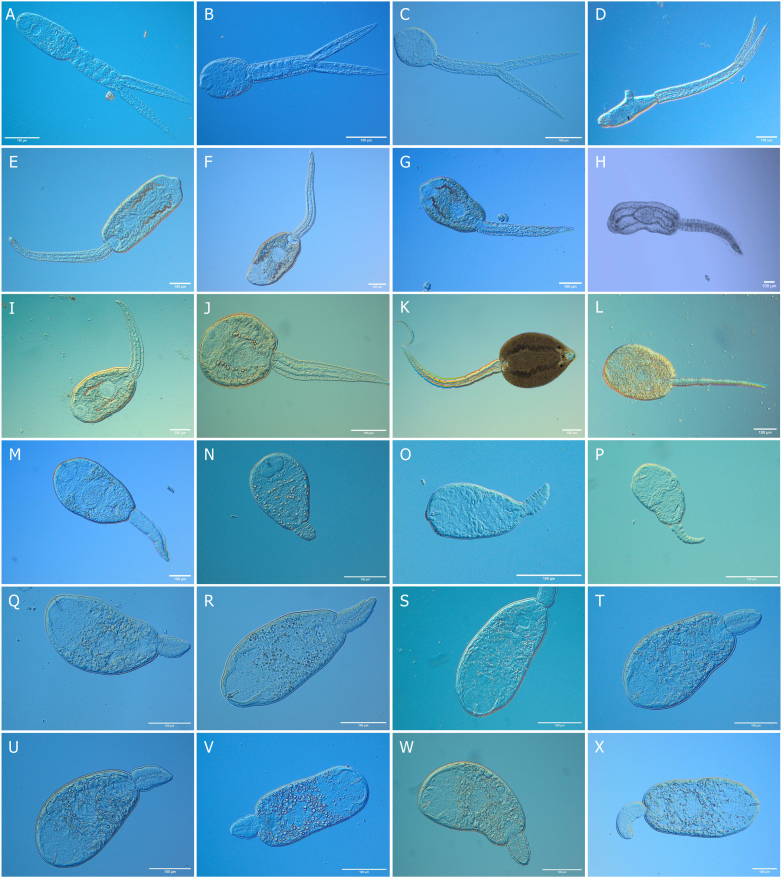
Photomicrographs of live cercariae of the identified trematode species: *Apatemon gracilis* (**A**), *Australapatemon burti* (**B**), *Cotylurus* sp. (**C**), *Trichobilharzia* sp. (**D**), *Echinoparyphium recurvatum* (**E**), *Echinostoma revolutum* (**F**), *Hypoderaeum conoideum* (**G**), *Moliniella anceps* (**H**), *Sphaeridiotrema* sp. OK-2019 (**I**), *Sphaeridiotrema pseudoglobulus* (**J**), *Notocotylus* sp. AK-2017 (**K**), *Opisthioglyphe ranae* (**L**), *Cephalogonimus* sp. EM-2024 (**M**), *Leyogonimus polyoon* (**N**), *Prosthogonimus ovatus* (**O**), *Lecithodendrium linstowi* (**P**), *Plagiorchis elegans* (**Q**), *Plagiorchis koreanus* (**R**), *Plagiorchis muelleri* (**S**), *Plagiorchis* sp. 3 (**T**), *Plagiorchis* sp. 7 (**U**), *Plagiorchis* sp. 2 (**V**), *Plagiorchis vespertilionis* (**W**), *Lecithopyge* sp. BO-2024 (**X**).

Trematode taxa were identified using an integrative approach combining cercarial morphology, sequence similarity to GenBank records (all isolates), and phylogenetic placement of newly generated molecular vouchers (subset of representative isolates). Infections for which molecular data could not be obtained were morphologically identified to genus level only (*Plagiorchis* sp., *Notocotylus* sp.; Table 2). In total, we identified 25 distinct trematode species from 11 families (Fig. 3, Table 2). Trematode infections were detected in five snail species (*A. balthica*, *B. tentaculata*, *L. stagnalis*, *P. carinatus*, and *S. palustris*), with an overall prevalence of 18.9%. *Ampullaceana balthica* harbored the highest trematode richness (17 species) and overall prevalence (28.0%) in the catchment (Fig. 2, Table 2). Overlap in infections among snail hosts was observed for several trematode taxa, including *Australapatemon burti* and *Echinoparyphium recurvatum*, which occurred in all lymnaeid snails as well as the planorbid *P. carinatus*. *Bithynia tentaculata* harbored a distinct trematode fauna consisting of five species from the families Lecithodendriidae, Pleurogenidae, Prosthogonimidae, and Psilostomidae, that were not detected in any other host species (Fig. 2). Co-infections with two trematode species were recorded from ten snails (all *A. balthica*, Supplementary file 2: Table S5) and were treated as separate infections in subsequent analyses. The most species-rich trematode families were Plagiorchiidae (8 species), Echinostomatidae (5 species), and Strigeidae (3 species). The most abundant trematode taxa were *E. recurvatum* (431 infections), *Notocotylus* sp. AK-2017 *sensu* Soldánová et al. (2017) (162 infections) and *A. burti* (152 infections). Life cycle reconstructions indicated that trematodes using waterfowl as definitive hosts were the most species-rich group (9 species), followed by bats (4 species) and amphibians (3 species). For four species, no definitive host group could be assigned (Table 2).

### 3.2 Environmental factors shaping trematode species richness and overall prevalence

The full model outputs and diagnostics are presented in Supplementary file 4. Including data from all snail species, recently restored sites did not differ significantly from mature or unimpacted sites in both trematode richness (*P* = 0.876 and *P* = 0.515, respectively) and overall prevalence (*P* = 0.843 and *P* = 0.281, respectively). Urban land use was associated with both significantly lower trematode richness (*β* = −0.88, *P* = 0.042) and overall prevalence (*β* = −1.36, *P* = 0.043) compared to agricultural sites, while no significant differences were reported for the remaining land use combinations. In addition, higher snail richness was negatively associated (*β* = −0.15, *P* = 0.004) with overall prevalence, whereas dissolved oxygen showed a positive relationship (*β* = 0.18, *P* = 0.010). Model estimates further suggested lower overall prevalence in winter compared to spring (*β* = −0.92, *P* = 0.020); however, this seasonal difference was not statistically significant after adjustment for multiple comparisons using Tukey’s HSD (*P* = 0.096).

For *A. balthica* snails, recently restored sites did not differ significantly from mature or unimpacted sites in trematode richness (*P* = 0.658 and *P* = 0.657, respectively) or overall prevalence (*P* = 0.628 and *P* = 0.517, respectively). Trematode richness did not show differences between urban land use, agricultural or forested sites (*P* = 0.190 and *P* = 0.996, respectively), but was associated with significantly lower overall prevalence than agricultural sites (*β* = −1.18, *P* = 0.005). Agricultural sites were further positively associated with higher trematode richness than forested sites (*P* = 0.049). Mean snail size was positively associated with both higher trematode richness (*β* = 0.22, *P* = 0.003) and overall prevalence (*β* = 0.77, *P* < 0.001).

**Fig. 4:**
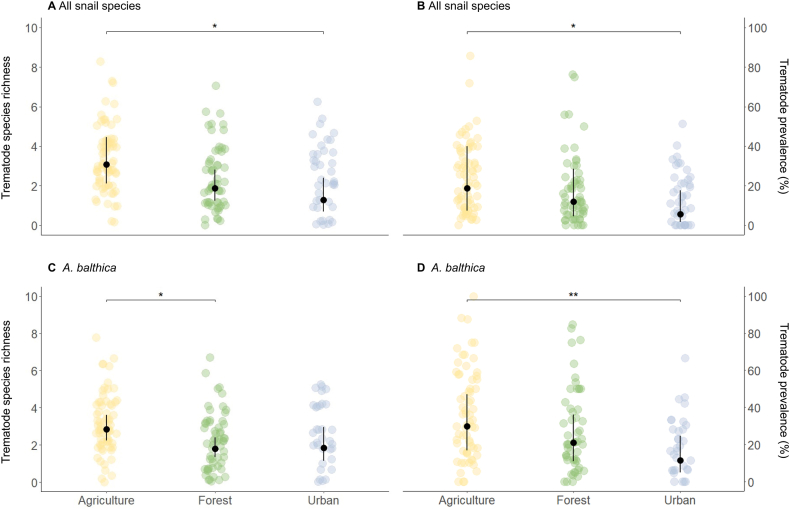
Estimated marginal means (EMMs; black dots ± standard error, SE) of trematode species richness and overall prevalence across land-use types for all snail species (**A**, **B**) and for *Ampullaceana balthica* (**C**, **D**). Points represent individual site-level observations. Predictions are back-transformed to the response scale. Asterisks indicate significant pairwise differences (∗*P* < 0.05, ∗∗*P* < 0.01).

In all models, assessment of patent trematode infections only (MRR) was associated with significantly lower detected overall prevalence, but not with detected species richness. As land use was identified as a reoccurring significant predictor, estimated marginal means (EMMs) were back-transformed to the response scale and plotted, highlighting lower predicted trematode richness and overall prevalence at urban and forested sites compared to agricultural sites (Fig. 4).

### 3.3 Environmental factors shaping trematode component communities

The full model outputs and diagnostics are presented in Supplementary file 4. Shannon diversity did not differ significantly across site groups (recently restored *vs* mature: *P* = 0.534; recently restored *vs* unimpacted: *P* = 0.970; mature *vs* unimpacted: *P* = 0.778). Urban sites did not differ significantly from either agricultural or forested sites (*P* = 0.974 and *P* = 0.146, respectively), but higher diversity was observed at agricultural compared to forested sites (*P* = 0.015). Shannon diversity further differed significantly among host species, with significantly higher diversity in *A. balthica* (Supplementary file 4: Table S21). No differences were observed for water parameters, the assessment of trematode infections (MRR *vs* dissection), or between seasons.

**Fig. 5:**
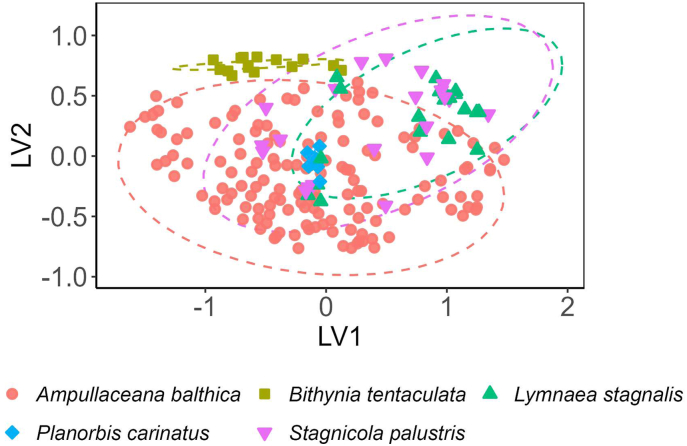
Unconstrained ordination of trematode component communities, with similarity based on the two-dimensional reduced-rank latent variables (LV1-LV2) derived from the generalized latent variable mixed model (GLVMM) on trematode abundance. Colored symbols represent component communities indicated by host species. Dashed ellipses show 95% confidence regions for host-specific centroids.

Community composition was investigated based on trematode abundance (given as the number of infected hosts per trematode species). Neither site group (recently restored *vs* mature: *P* = 0.919; recently restored *vs* unimpacted: *P* = 0.583; mature *vs* unimpacted: *P* = 0.759) nor land use (urban *vs* agriculture: *P* = 9.147; urban *vs* forest: *P* = 0.978; agriculture *vs* forest: *P* = 0.176) significantly influenced component communities. However, significant differences in community composition were observed among host species (Fig. 5, Supplementary file 4: Table S22), and for snail host size (*P* < 0.001). No significant differences were observed for water parameters or the assessment method of trematode infections.

### 3.4 Transmission pathways in different land use types

**Fig. 6:**
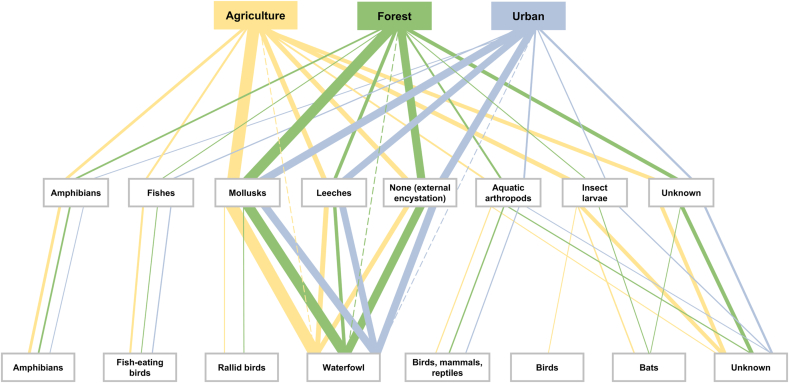
Transmission pathways of trematode species from their first intermediate snail hosts collected at sites with different land use types (agriculture, forest, urban). Middle-row boxes indicate second intermediate host groups, and bottom-row boxes represent definitive host groups. Lines between boxes correspond to distinct trematode species utilizing specific transmission pathways, with line thickness proportional to the overall prevalence of species using the respective transmission route. The dashed lines show direct infection of the definitive host.

To explore how trematode distribution and prevalence varied across land use types, we further analyzed life cycle dynamics of the identified species (Fig. 6). As trematode communities did not differ significantly across land-use types, this was reflected in similar definitive host groups among sites. However, species infecting rallid birds and bats exclusively occurred at agricultural and forested sites, and *P. ovatus* infecting birds was found exclusively at agricultural sites. Trematodes infecting waterfowl as definitive hosts predominated at all land-use types, but overall prevalence patterns differed by transmission route. Species transmitted to waterfowl *via* molluscs as second intermediate hosts were most prevalent at agricultural sites (overall prevalence of 7.9%), followed by forested (6.6%) and urban sites (4.8%). Trematodes infecting amphibians and fish-eating birds were likewise most frequent at agricultural sites, with an overall prevalence of 1.4% (forested 0.7%; urban 0.1%), and 0.9% (forested 0.1%; urban 0.3%), respectively. In contrast, trematodes infecting waterfowl *via* truncated life cycles with external encystation of metacercariae were most prevalent at forested (5.2%) and urban sites (4.7%), compared to 3.1% at agricultural sites. Overall prevalence of trematodes transmitted to waterfowl *via* leeches peaked at urban sites (4.2% *vs* 3.0% at agricultural and 1.8% at forested sites). Among the species for which life cycles could be inferred, all featured allogenic life cycles with definitive hosts capable of moving between aquatic and terrestrial habitats, whereas we detected no autogenic taxa completing their life cycle entirely within the aquatic system.

## 4 Discussion

This study provides a fine-scale assessment of trematode infection dynamics across an urban stream catchment, based on two years of sampling 6300 snails and 25 trematode species from 11 families. We detected four taxa which represent novel regional records, including an unidentified species Echinostomatidae gen. sp. closely related to Echinostomatidae gen. sp. OK-2019 *sensu* Pantoja et al. (2021), and *Plagiorchis* sp. 2, 3, and 7 *sensu* Soldánová et al. (2017), previously reported from Norway, Finland, and the Czech Republic (Soldánová et al., 2017; Kudlai et al., 2021; Kundid et al., 2024). By integrating restoration context, adjacent land use, and host-related factors shaping trematode infection in first intermediate snail hosts, we identified dominant drivers of trematode diversity and infection patterns across this urban catchment.

Despite the distinct history of degradation and subsequent restoration in the Boye catchment, restoration history of sites did not predict trematode species richness or overall prevalence (H1). Recently restored sites (< 5 years prior to sampling) supported trematode richness and overall prevalence comparable to sites restored more than two decades ago or sites never impacted by wastewater input, suggesting rapid recolonization of habitats by both susceptible snails and definitive hosts. Similarly, quick recovery of habitats has been documented in estuarine and coastal restoration contexts where trematode diversity and prevalence were restored within approximately six years, presumably driven by host dispersal of bird definitive hosts (Huspeni and Lafferty, 2004; Aguirre-Macedo et al., 2011). Trematodes can broadly be differentiated based on the dispersal capacity of their definitive host, with autogenic species completing their life cycle entirely within aquatic systems, whereas allogenic species require at least one host capable of moving between aquatic and terrestrial habitats, such as birds or mammals (Esch et al., 1988). Among the taxa for which life cycles could be reconstructed, we exclusively detected allogenic trematodes in the Boye catchment, with many species transmitted by waterfowl definitive hosts that can likely facilitate recolonization and transmission shortly after restoration. Nevertheless, autogenic trematodes can also recover quickly when intermediate and definitive hosts recolonize efficiently, as shown by Moore et al. (2020), who observed increases in fish and crustacean communities and their parasites within one year of restoration. The widespread occurrence and high abundance of *A. balthica* across the catchment may have further promoted the quick establishment of trematode communities, as this species was identified as a key host that harbored the majority of detected trematode species and the highest infection prevalence. *Ampullaceana balthica* is known for low habitat requirements and high susceptibility to diverse trematode taxa, and consequently often supports species-rich trematode assemblages where established (Soldánová et al., 2017; Schwelm et al., 2021; Kundid et al., 2024; Faltýnková et al., 2025).

In contrast to restoration history, adjacent land use consistently predicted trematode species richness and overall prevalence (H2), albeit with slight variation among snail hosts. While we expected lower trematode species richness and overall prevalence at urbanized sites, agricultural sites differed most strongly from both urban and forested habitats, supporting higher trematode species richness and overall prevalence. Although agricultural land use is generally associated with negative effects on in-stream freshwater biodiversity (Schürings et al., 2022), agricultural stream reaches may support high densities of first intermediate snail hosts (Johnson et al., 2007; Altman and Byers, 2014). In addition, these sites may enhance accessibility and attractiveness for definitive hosts in the system by providing open sightlines and diverse edge habitats (Heim et al., 2015; Fox et al., 2017; Finch et al., 2020). Within the heterogeneous landscape of the Boye catchment, agricultural sites might thus combine comparatively lower levels of direct anthropogenic disturbance with structural features favorable to host foraging and movement, leading to higher frequentation by definitive hosts than either urban or forested sites. Variation in habitat use of these hosts may, in turn, facilitate elevated trematode transmission and result in increased overall prevalence and species richness (Hechinger and Lafferty, 2005). Interpretation of the observed land-use effects should consider that both urban sites in our study were recently restored, risking confounding land-use and restoration effects for this category. Nevertheless, the two remaining recently restored sites represented agricultural and forested land use, respectively, and exhibited significantly higher trematode richness and overall prevalence than their urban counterparts, suggesting that land-use effects were a stronger predictor of trematode dynamics than restoration history in our dataset.

We observed no substantial shifts in trematode component community composition across restoration history or land-use types (H3), apart from higher Shannon diversity in agricultural compared to forested habitats. This indicates limited species turnover across the catchment and suggests that local differences among sites primarily reflect changes in species richness or prevalence within broadly similar trematode communities. These patterns may result from the observed dominance of allogenic taxa dispersed *via* mobile definitive hosts, facilitating trematode dispersal among sites and thus promoting homogenization of trematode assemblages. While shifts in community composition have been documented as a response to environmental drivers such as nutrient input (Esch et al., 1988) or agricultural land use (Hammoud et al., 2025), the lack of differentiation among community composition in our study suggests that landscape-level processes, such as host movement, may override local habitat differences in structuring trematode communities in the Boye catchment. At the same time, Shannon diversity and trematode component communities substantially differed among first intermediate host species, highlighting that local trematode assemblages cannot be interpreted independently of intermediate host availability, as trematodes typically exhibit high specificity to the gastropod host (Esch et al., 2001). This was especially pronounced for *B. tentaculata*, which harbored a distinct trematode assemblage with no overlap to other snail hosts, in line with previous studies on this species (Schwelm et al., 2020; Faltýnková et al., 2024). In addition, component communities varied with mean snail size, consistent with the expectation that larger (and thus older) snails accumulate infections over time and therefore reflect a different exposure history than smaller individuals (Graham, 2003; Tolley-Jordan and Chadwick, 2019).

We detected no significant seasonal patterns in trematode richness, prevalence, or community composition, suggesting limited temporal variation in overall transmission dynamics across the sampling period. This may reflect continuous availability of definitive hosts throughout the year, as is the case for many species of waterfowl in temperate Europe (Frost et al., 2019; Selbach et al., 2020). In addition, assessment of patent infections only (MRR) resulted in lower estimates of overall trematode prevalence but did not reduce detected species richness or shift community composition compared to dissection of snails. While previous studies have already demonstrated that assessing patent infections only does not accurately reflect prevalence (e.g. Born-Torrijos et al., 2014; Farghaly et al., 2016), our data suggest that surveys based on patent infections can nonetheless provide a robust representation of trematode communities.

Trematodes have been proposed as promising bioindicators of ecosystem condition, as their complex life cycles can reflect the presence and diversity of free-living hosts, including rare or protected species (Sures et al., 2017; Schwelm et al., 2021; Hüsken et al., 2025). Our findings suggest that diverse trematode communities can establish rapidly in restored habitats, supporting their potential for assessing ecological recovery and succession in freshwater systems. However, the lack of consistent restoration effects also indicates that trematode responses to environmental drivers may be strongly context dependent and should be interpreted alongside drivers such as land use and the distribution of key intermediate host species.

## 5 Conclusion

Adjacent land use emerged as a consistent driver of trematode dynamics in our study, with agricultural sites exhibiting higher species richness and overall prevalence. However, these patterns were not reflected by shifts in trematode community composition, suggesting that catchment-wide dispersal mediated by mobile definitive hosts may homogenize assemblages despite local variation in richness and prevalence. Restoration history did not predict trematode dynamics, consistent with a rapid recovery of trematode communities in recently restored habitats. Finally, the trematode diversity documented in this urban stream system highlights the importance of key host species in sustaining diverse and abundant trematode communities within anthropogenically degraded and restored habitats.

## Ethical approval

Not applicable.

## CRediT authorship contribution statement

**Annabell Hüsken:** Writing – original draft, Writing – review & editing, Investigation, Visualization, Methodology, Formal analysis. **Jessica Schwelm:** Writing – review & editing, Methodology, Resources, Supervision. **Bernd Sures:** Writing – review & editing, Conceptualization, Resources, Supervision, Funding acquisition.

## Funding

This study was performed within the Collaborative Research Center (10.13039/501100003383CRC) RESIST (A09) funded by the 10.13039/501100001659Deutsche Forschungsgemeinschaft (10.13039/501100001659DFG, 10.13039/501100001659German Research Foundation) - 10.13039/501100003383CRC 1439/1 and 1439/2 - project number: 426547801.

## Declaration of competing interests

The authors declare that they have no known competing financial interests or personal relationships that could have appeared to influence the work reported in this paper.

## Data Availability

All data generated and analyzed during this study are included in this article or available from Zenodo (https://doi.org/10.5281/zenodo.18411766). Subsets of these data have already been published and have been referenced accordingly (Hüsken et al., 2025). The raw data that support the present findings are part of an ongoing study and can be made available from the corresponding author upon request.
