# Supplementary material for: Land use drives trematode dynamics in a restored stream system
Source: Curr Res Parasitol Vector Borne Dis. 2026 Feb 13;9:100357. doi: 10.1016/j.crpvbd.2026.100357 (PMC12954508; doi:10.1016/j.crpvbd.2026.100357)
Supplement: Multimedia component 2 [file mmc2.pdf]

Supplementary file 2

Supplementary Table S2. Primers used in this study.

**Gene marker**	**Primer name**	**Fragment length (bp)**	**Nucleotide sequence (5’–3’)**	**Reference**
Trematodes				
28S	digl2	~1200	AAG CAT ATC ACT AAG CGG	[76]
	1500R		GCT ATC CTG AGG GAA ACT TCG	[77]
	300F*		CAA GTA CCG TGA GGG AAA GTT G	[78]
	ECD2*		CCT TGG TCC GTG TTT CAA GAC GGG	[79]
*cox*1	JB3	~400	TTT TTT GGG CAT CCT GAG GTT TAT	[80]
	JB4.5		TAA AGA AAG AAC ATA ATG AAA ATG	
*nad*1	NDJ11	~500	AGA TTC GTA AGG GGC CTA ATA	[81]
	NDJ2a		CTT CAG CCT CAG CAT AAT	
Snails				
28S	F63.2	~1100	ACC CGC TGA AYT TAA GCA TA	[82]
	LSU3		TCC TGA GGG AAA CTT CGG	
*cox*1	LCO1490	~658	GGT CAA CAA ATC ATA AAG ATA TTG G	[83]
	HCO2198		TAA ACT TCA GGG TGA CCA AAA AAT CA	

*Sequencing primer.

Supplementary Table S3. Details on the sequence alignments used in the phylogenetic analyses for this study.

Trematode family	Gene region	Alignment number	No. of newly generated sequences	No. of GenBank sequences	Alignment length	Model ML	Model BI	Figure
Cephalogonimidae, Telorchiidae	28S	1	3	11	1108	TVM+F+G4	GTR+G	S1
Echinostomatidae	28S	2	13	19	985	GTR+F+I+G4	GTR+I+G	S2
	*nad*1	3	16	20	413	TIM+F+I+G4	GTR+I+G	S3
Notocotylidae	28S	4	5	20	1130	TVM+F+I+G4	GTR+I+G	S4
Plagiorchiidae	28S	5	20	26	1119	GTR+F+R3	GTR+I+G	S5
	*cox*1	6	19	19	323	TIM2+F+I+R2	GTR+I+G	S6
Lecithodendriidae, Pleurogenidae, Prosthogonimidae	28S	7	7	20	1055	GTR+F+I+R2	GTR+I+G	S7
Psilostomidae	28S	8	5	18	1045	GTR+F+R2	GTR+G	S8
Schistosomatidae	28S	9	3	19	923	GTR+F+G4	GTR+G	S9
	*cox*1	10	3	17	295	TPM2u+F+I+G4	GTR+I+G	S10
Strigeidae	28S	11	10	18	1049	TVM+F+G4	GTR+G	S11

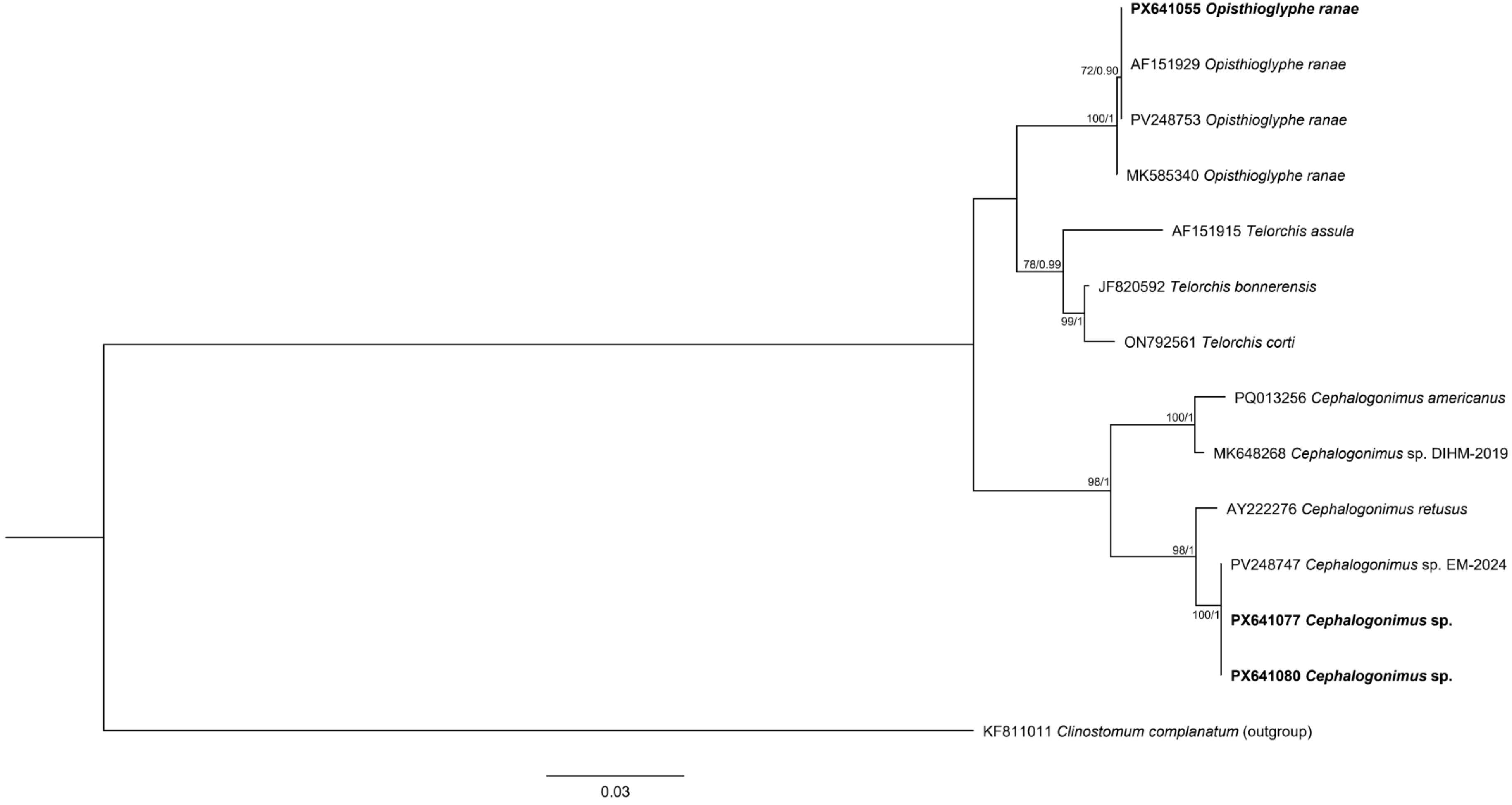

Supplementary Figure S1. Maximum likelihood (ML) phylogram based on Alignment 1 (partial 28S rDNA) for families Cephalogonimidae and Telorchiidae. Node support is indicated by bootstrap values from ML analysis and posterior probabilities from Bayesian inference (BI) analysis. Only values > 70 (ML) and 0.90 (BI) are displayed. Scale bar indicates the expected number of substitutions per site. Sequences generated in this study are presented in bold.

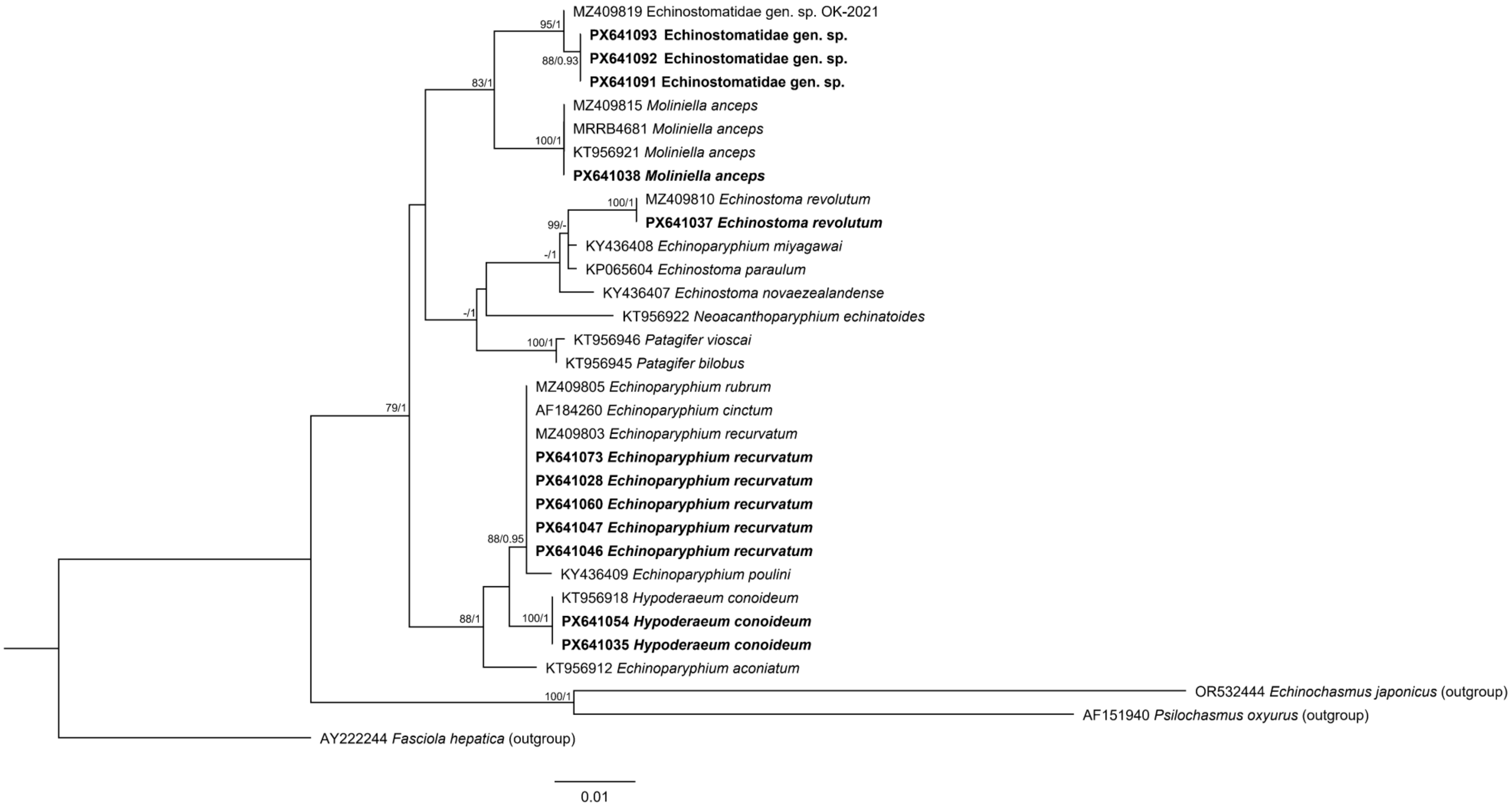

Supplementary Figure S2. Maximum likelihood (ML) phylogram based on Alignment 2 (partial 28S rDNA) for family Echinostomatidae. Node support is indicated by bootstrap values from ML analysis and posterior probabilities from Bayesian inference (BI) analysis. Only values > 70 (ML) and 0.90 (BI) are displayed. Scale bar indicates the expected number of substitutions per site. Sequences generated in this study are presented in bold.

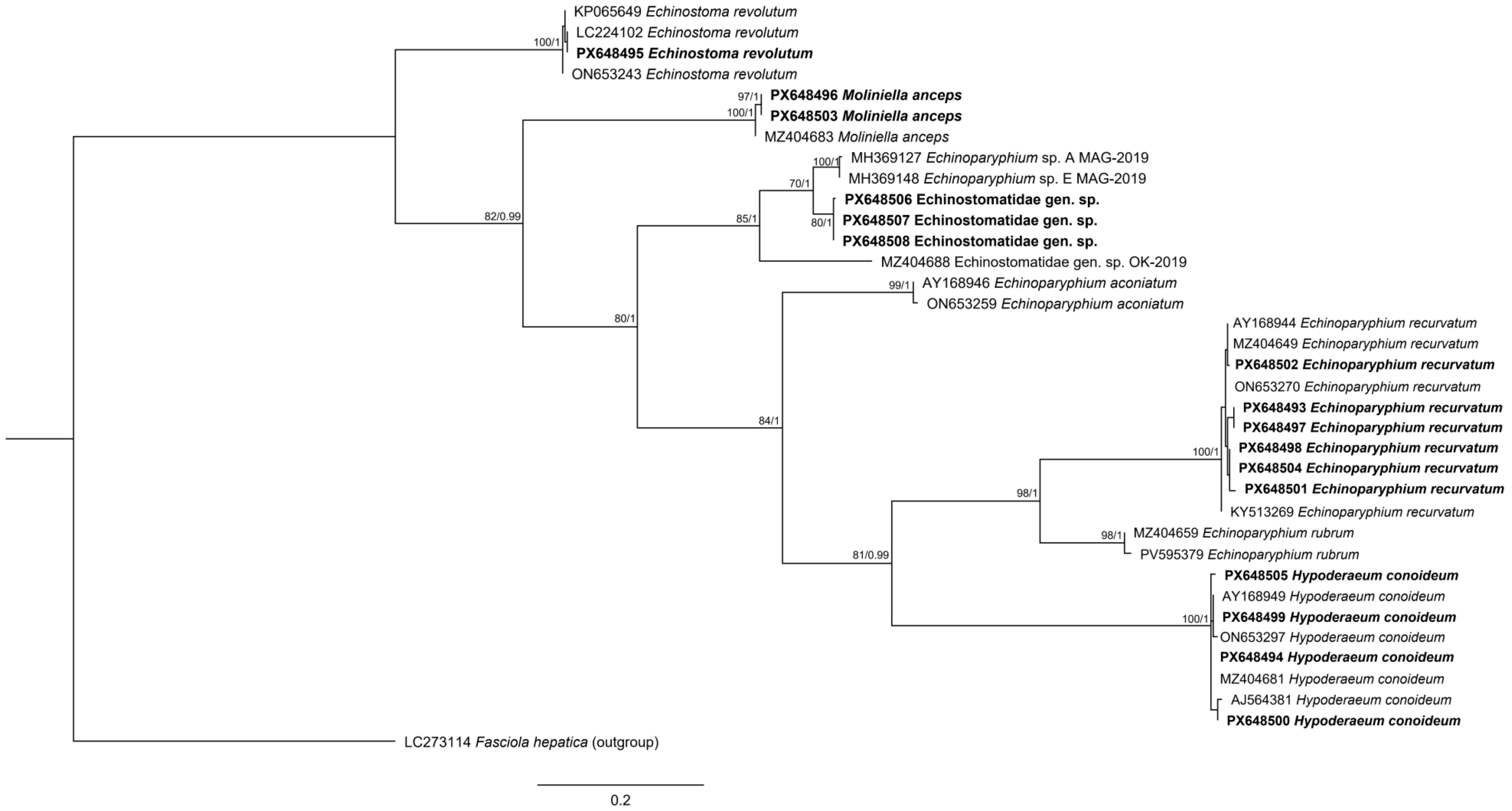

Supplementary Figure S3. Maximum likelihood (ML) phylogram based on Alignment 3 (*nad*1) for family Echinostomatidae. Node support is indicated by bootstrap values from ML analysis and posterior probabilities from Bayesian inference (BI) analysis. Only values > 70 (ML) and 0.90 (BI) are displayed. Scale bar indicates the expected number of substitutions per site. Sequences generated in this study are presented in bold.

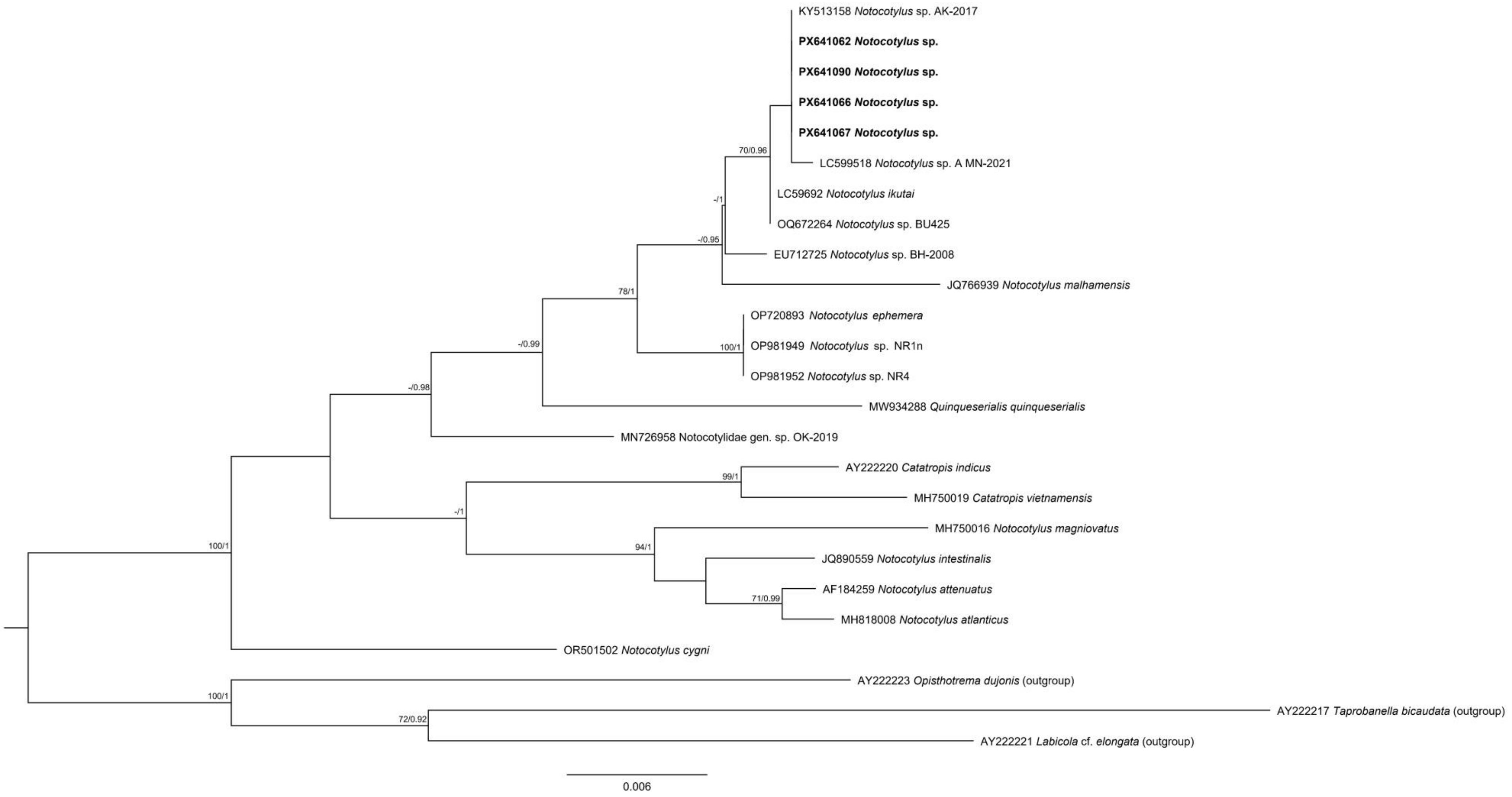

Supplementary Figure S4. Maximum likelihood (ML) phylogram based on Alignment 4 (partial 28S rDNA) for family Notocotylidae. Node support is indicated by bootstrap values from ML analysis and posterior probabilities from Bayesian inference (BI) analysis. Only values > 70 (ML) and 0.90 (BI) are displayed. Scale bar indicates the expected number of substitutions per site. Sequences generated in this study are presented in bold.

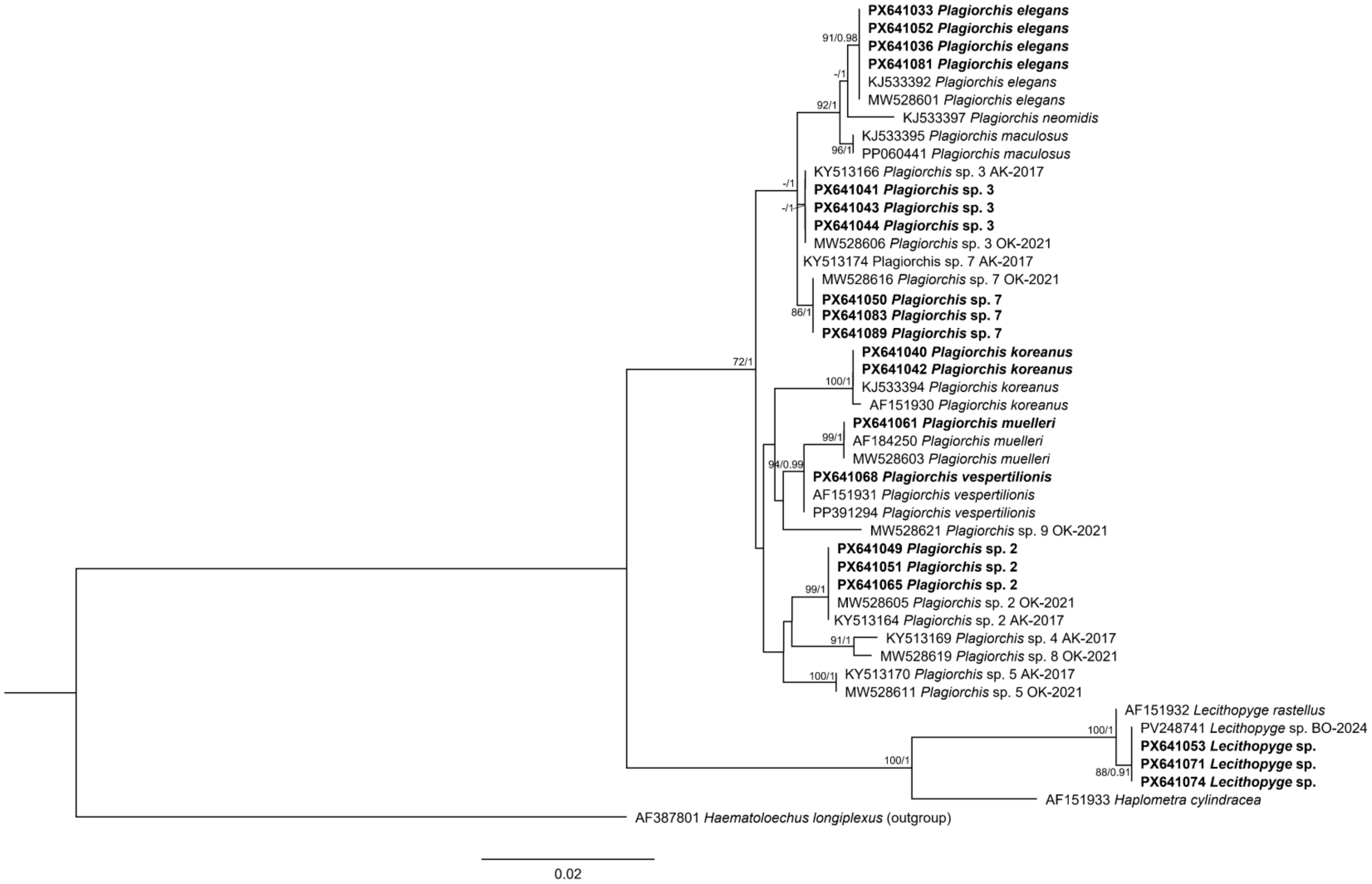

Supplementary Figure S5. Maximum likelihood (ML) phylogram based on Alignment 5 (partial 28S rDNA) for family Plagiorchiidae. Node support is indicated by bootstrap values from ML analysis and posterior probabilities from Bayesian inference (BI) analysis. Only values > 70 (ML) and 0.90 (BI) are displayed. Scale bar indicates the expected number of substitutions per site. Sequences generated in this study are presented in bold.

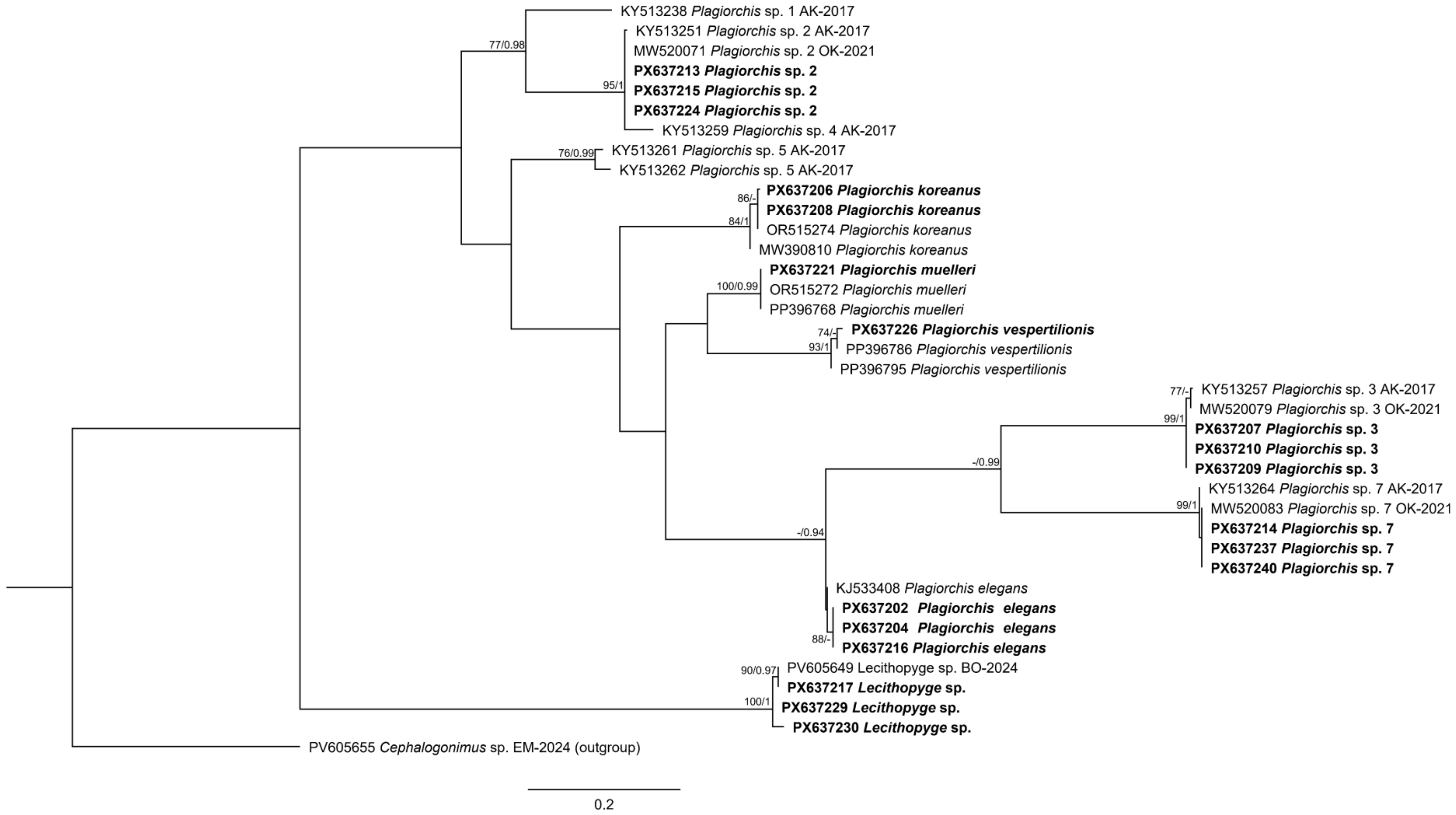

Supplementary Figure S6. Maximum likelihood (ML) phylogram based on Alignment 6 (*cox*1) for family Plagiorchiidae. Node support is indicated by bootstrap values from ML analysis and posterior probabilities from Bayesian inference (BI) analysis. Only values > 70 (ML) and 0.90 (BI) are displayed. Scale bar indicates the expected number of substitutions per site. Sequences generated in this study are presented in bold.

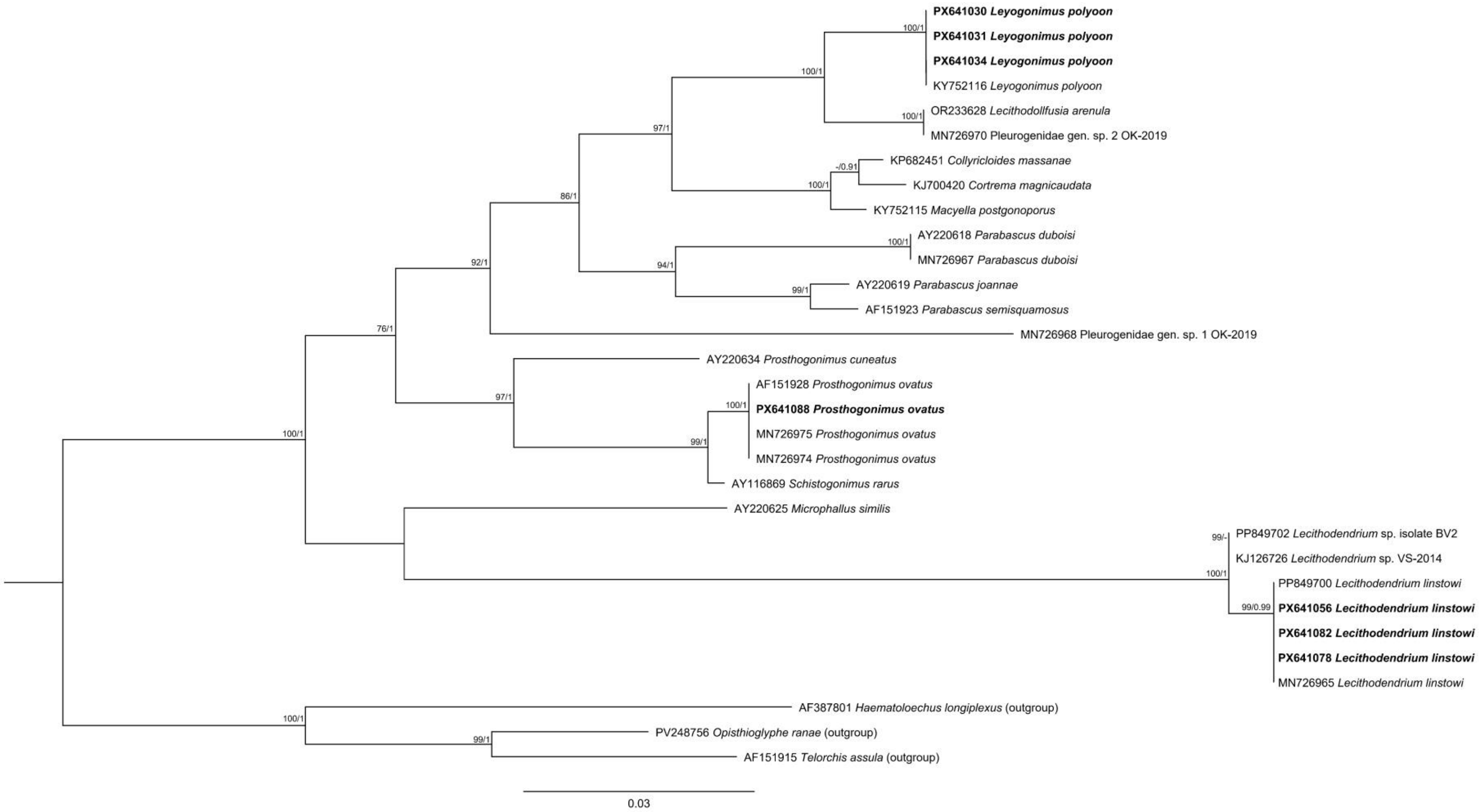

Supplementary Figure S7. Maximum likelihood (ML) phylogram based on Alignment 7 (partial 28S rDNA) for families Lecithodendriidae, Pleurogenidae, and Prosthogonimidae. Node support is indicated by bootstrap values from ML analysis and posterior probabilities from Bayesian inference (BI) analysis. Only values > 70 (ML) and 0.90 (BI) are displayed. Scale bar indicates the expected number of substitutions per site. Sequences generated in this study are presented in bold.

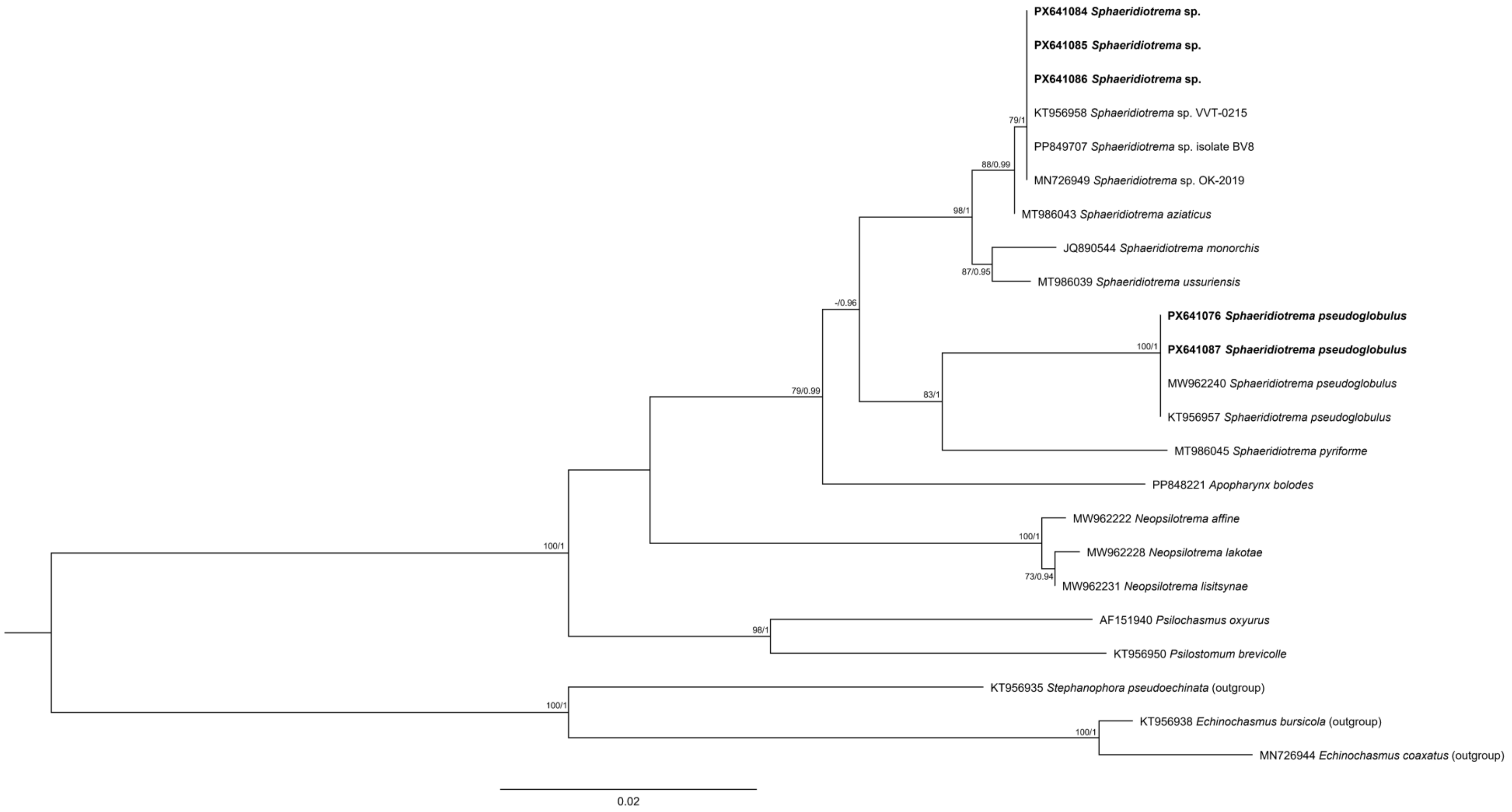

Supplementary Figure S8. Maximum likelihood (ML) phylogram based on Alignment 8 (partial 28S rDNA) for family Psilostomidae. Node support is indicated by bootstrap values from ML analysis and posterior probabilities from Bayesian inference (BI) analysis. Only values > 70 (ML) and 0.90 (BI) are displayed. Scale bar indicates the expected number of substitutions per site. Sequences generated in this study are presented in bold.

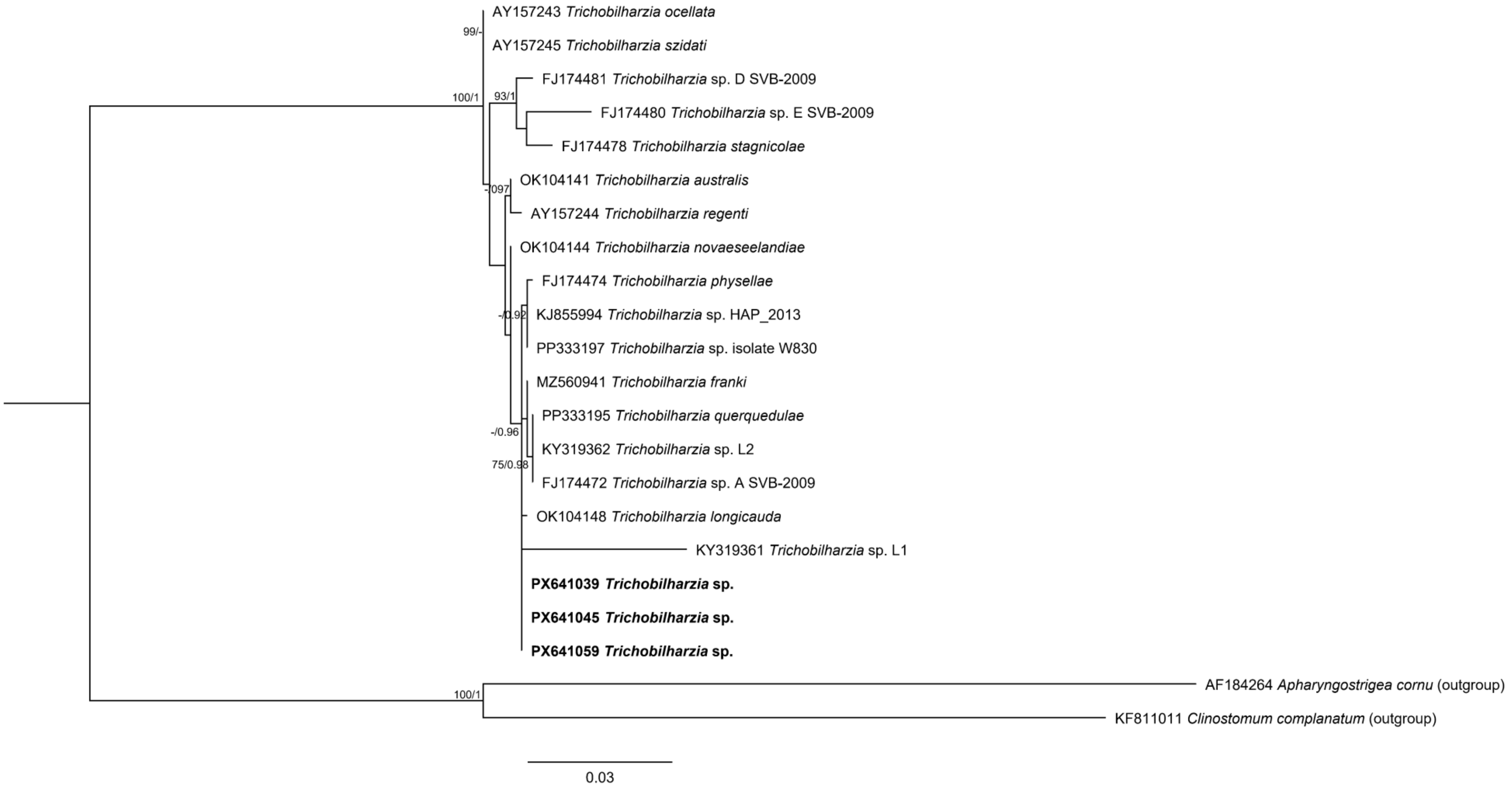

Supplementary Figure S9. Maximum likelihood (ML) phylogram based on Alignment 9 (28S) for family Schistosomatidae. Node support is indicated by bootstrap values from ML analysis and posterior probabilities from Bayesian inference (BI) analysis. Only values > 70 (ML) and 0.90 (BI) are displayed. Scale bar indicates the expected number of substitutions per site. Sequences generated in this study are presented in bold.

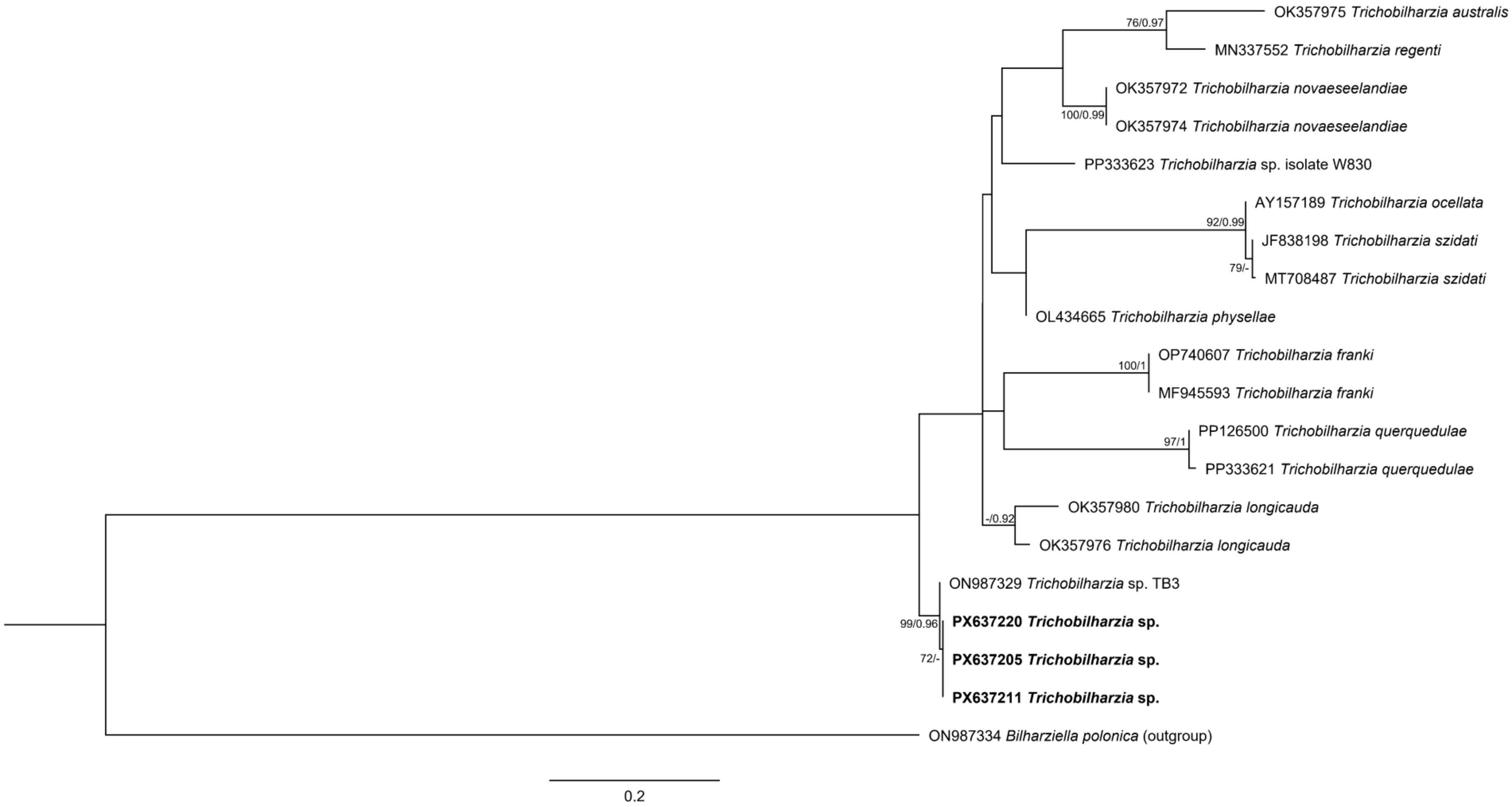

Supplementary Figure S10. Maximum likelihood (ML) phylogram based on Alignment 10 (*cox*1) for family Schistosomatidae. Node support is indicated by bootstrap values from ML analysis and posterior probabilities from Bayesian inference (BI) analysis. Only values > 70 (ML) and 0.90 (BI) are displayed. Scale bar indicates the expected number of substitutions per site. Sequences generated in this study are presented in bold.

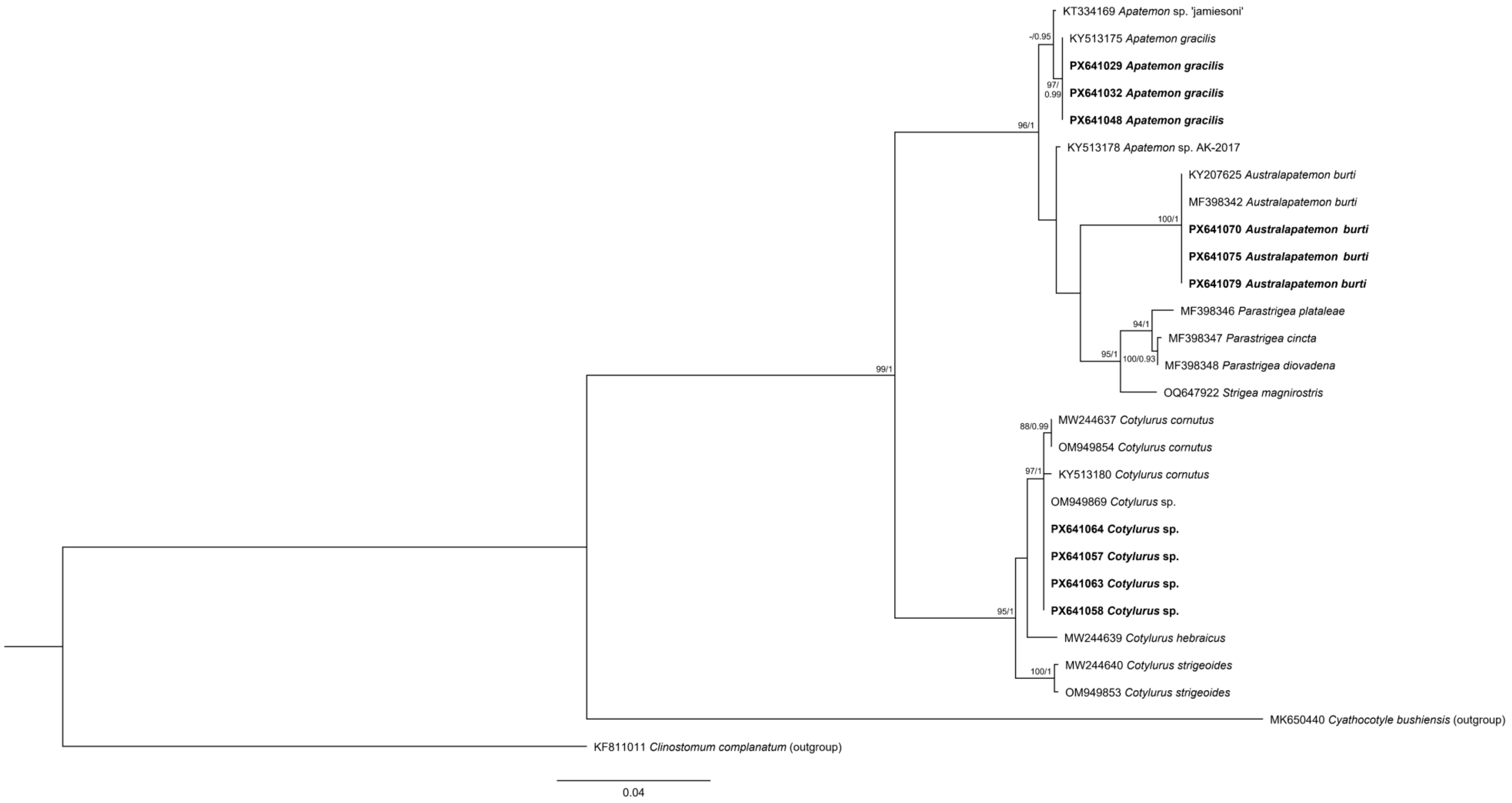

Supplementary Figure S11. Maximum likelihood (ML) phylogram based on Alignment 11 (partial 28S rDNA) for family Strigeidae. Node support is indicated by bootstrap values from ML analysis and posterior probabilities from Bayesian inference (BI) analysis. Only values > 70 (ML) and 0.90 (BI) are displayed. Scale bar indicates the expected number of substitutions per site. Sequences generated in this study are presented in bold.

Supplementary Table S4. Summary of all sequences of trematodes used for 28S and *cox*1/*nad*1 phylogenetic analyses. The novel sequences generated in this study are highlighted in bold. Life cycle stage: A, adult; S, sporocyst; C, cercaria; MC, metacercaria; J, juvenile.

Taxon	Life cycle stage	Host	Country	GenBank Accession No.		Reference
				28S	*cox*1/*nad*1*	
**Family Cephalogonimidae**						
Cephalogonimus americanus	C	*Planorbella trivolis*	USA	PQ013256	–	[1]
Cephalogonimus retusus	A	*Pelophylax ridibundus*	Bulgaria	AY222276	–	[2]
Cephalogonimus sp. DIHM-2019	A	*Lithobathes* sp.	Mexico	MK648268	–	[3]
Cephalogonimus sp. EM-2024	C	*Ampullaceana balthica*	Germany	PV248747	PV605655	[4]
***Cephalogonimus* sp.**	**C**	***Ampullaceana balthica***	**Germany**	**PX641077**	**PX637233**	**This study**
***Cephalogonimus* sp.**	**C**	***Ampullaceana balthica***	**Germany**	**PX641080**	**PX637235**	**This study**
**Family Clinostomidae**						
Clinostomum complanatum	MC	*Heteropneustes fossilis*	India	KF811011	–	[5]
**Family Cathocotylidae**						
Cyathocotyle bushiensis	A	*Aythya affinis*	USA	MK650440	–	[6]
**Family Echinochasmidae**						
Echinochasmus bursicola	A	*Ardea alba*	Ukraine	KT956938	–	[7]
Echinochasmus coaxatus	C	*Bithynia tentaculata*	Germany	MN726944	–	[8]
Echinochasmus japonicus	A	*Homo sapiens*	Vietnam	OR532444	–	[9]
Stephanoprora pseudoechinata	A	*Chroicocephalus genei*	Ukraine	KT956935	–	[7]
**Family Echinostomatidae**						
Echinoparyphium aconiatum	C	*Lymnaea stagnalis*	Czech Republic	KT956912	–	[7]
Echinoparyphium aconiatum	C	*Lymnaea stagnalis*	Finland	–	AY168946	[10]
Echinoparyphium aconiatum	C	*Lymnaea stagnalis*	UK	–	ON653259	[11]
Echinoparyphium cinctum	A	*Anas platyrhynchos*	Ukraine	AF184260	–	[12]
Echinoparyphium poulini	A	*Cygnus atratus*	New Zealand	KY436409	–	[13]
Echinoparyphium recurvatum	C	*Myxas glutinosa*	Finland	MZ409803	–	[14]
Echinoparyphium recurvatum	C	*Lymnaea peregra*	UK	–	AY168944	[10]

Echinoparyphium recurvatum	C	*Ampullaceana balthica*	Iceland	–	MZ404649	[14]
Echinoparyphium recurvatum	C	*Radix auricularia*	UK	–	ON653270	[11]
Echinoparyphium recurvatum	MC	*Sphaerium* sp.	Norway	–	KY513269	[13]
Echinoparyphium recurvatum	**C**	***Ampullaceana balthica***	**Germany**	**PX641060**	**PX648501***	**This study**
Echinoparyphium recurvatum	**C**	***Ampullaceana balthica***	**Germany**	**PX641028**	**PX648493***	**This study**
Echinoparyphium recurvatum	**C**	***Ampullaceana balthica***	**Germany**	**PX641073**	**PX648504***	**This study**
Echinoparyphium recurvatum	**C**	***Stagnicola palustris***	**Germany**	**PX641046**	**PX648497***	**This study**
Echinoparyphium recurvatum	**C**	***Stagnicola palustris***	**Germany**	**PX641047**	**PX648498***	**This study**
Echinoparyphium recurvatum	**C**	***Planorbis carinatus***	**Germany**	–	**PX648502***	**This study**
Echinoparyphium rubrum	C	*Ladisvella elodes*	USA	MZ409805	MZ404659	[14]
Echinoparyphium rubrum	C	*Ladisvella elodes*	Canada	–	PV595379	[15]
Echinoparyphium sp. A MAG-2019	C	*Ladisvella elodes*	Canada	–	MH369127	[16]
Echinoparyphium sp. E MAG-2019	C	*Lymnaea stagnalis*	Canada	–	MH369148	[16]
Echinostoma miyagawai	J	*Anas platyrhynchos*	New Zealand	KY436408	–	[13]
Echinostoma novazealandese	A	*Cygnus atratus*	New Zealand	KY436407	–	[13]
Echinostoma paraulum	C	*Lymnaea stagnalis*	Germany	KP065604	–	[17]
Echinostoma revolutum	C	*Ampullaceana balthica*	Iceland	MZ409810	–	[14]
Echinostoma revolutum	C	*Lymnaea stagnalis*	Germany	–	KP065649	[17]
Echinostoma revolutum	A	*Anas platyrhynchos*	Bangladesh	–	LC224102	[18]
Echinostoma revolutum	C	*Stagnicola palustris*	UK	–	ON653243	[11]
Echinostoma revolutum	**C**	***Ampullaceana balthica***	**Germany**	**PX641037**	**PX648495***	**This study**
Echinostomatidae gen. sp. OK-2021	C	*Lymnaea stagnalis*	Finland	MZ409819	MZ404688	[14]
**Echinostomatidae gen. sp.**	**C**	***Ampullaceana balthica***	**Germany**	**PX641091**	**PX648506***	**This study**
**Echinostomatidae gen. sp.**	**C**	***Stagnicola palustris***	**Germany**	**PX641092**	**PX648507***	**This study**
**Echinostomatidae gen. sp.**	**C**	***Lymnaea stagnalis***	**Germany**	**PX641093**	**PX648508***	**This study**
Hypoderaeum conoideum	A	*Anas platyrhynchos*	Ukraine	KT956918	–	[7]
Hypoderaeum conoideum	C	*Radix auricularia*	UK	–	ON653297	[11]
Hypoderaeum conoideum	C	*Lymnaea peregra*	Bulgaria	–	AY168949	[10]
Hypoderaeum conoideum	C	*Lymnaea stagnalis*	Finland	–	MZ404681	[14]

Hypoderaeum conoideum	A	*Gallus gallus*	Spain	–	AJ564381	[19]
Hypoderaeum conoideum	**C**	***Lymnaea stagnalis***	**Germany**	**PX641035**	**PX648494***	**This study**
Hypoderaeum conoideum	**C**	***Ampullaceana balthica***	**Germany**	**PX641054**	**PX648499***	**This study**
Hypoderaeum conoideum	**C**	***Stagnicola palustris***	**Germany**	–	**PX648505***	**This study**
Hypoderaeum conoideum	**C**	***Ampullaceana balthica***	**Germany**	–	**PX648500***	**This study**
Moliniella anceps	MC	*Planorbarius corneus*	Lithuania	KT956921	–	[7]
Moliniella anceps	C	*Stagnicola fuscus*	Ireland	MZ409815	MZ404683	[14]
Moliniella anceps	**C**	***Stagnicola palustris***	**Germany**	**PX641038**	**PX648496***	**This study**
Moliniella anceps	**C**	***Stagnicola palustris***	**Germany**	**PX641072**	**PX648503***	**This study**
Neoacanthoparyphium echinatoides	C	*Viviparus acerosus*	Slovakia	KT956922	–	[7]
Patagifer bilobus	A	*Plegadis falcinellus*	Ukraine	KT956945	–	[7]
Patafiger vioscai	A	*Eudocimus albus*	USA	KT956946	–	[7]
**Family Fasciolidae**						
Fasciola hepatica	A	*Capra hircus*	Saudi Arabia	AY222244		[2]
Fasciola hepatica	A	*Bos taurus*	Ecuador	–	LC3114	[20]
**Family Haematoloechidae**						
Haematoloechus longiplexus	A	*Rana catesbeiana*	USA	AF387801	–	[21]
**Family Labicolidae**						
Labicola cf. *elongata*	A	*Dugong dugon*	Australia	AY222221	–	[2]
**Family Lecithodendriidae**						
Lecithodendrium linstowi	C	*Bithynia tentaculata*	Ireland	PP849700	–	[22]
Lecithodendrium linstowi	C	*Bithynia tentaculata*	Germany	MN726965	–	[8]
Lecithodendrium linstowi	**C**	***Bithynia tentaculata***	**Germany**	**PX641056**	**PX637218**	**This study**
Lecithodendrium linstowi	**C**	***Bithynia tentaculata***	**Germany**	**PX641078**	–	**This study**
Lecithodendrium linstowi	**C**	***Bithynia tentaculata***	**Germany**	–	**PX637234**	**This study**
Lecithodendrium linstowi	**C**	***Bithynia tentaculata***	**Germany**	**PX641082**	**PX637236**	**This study**
Lecithodendrium sp.	C	*Bithynia tentaculata*	Ireland	PP849702	–	[22]
Lecithodendrium sp. VS-2014	P	*Bithynia tentaculata*	Lithuania	KJ126726	–	[23]
**Family Microphallidae**						

Microphallus similis	A	*Carcinus maenas*	UK	AY220625	–	[24]
**Family Notocotylidae**						
Catatropis indicus	A	*Cairina moschata*	Australia	AY222220	–	[2]
Catatropis vietnamensis	A	*Anas platyrhynchos*	Vietnam	MH750019	–	[25]
Notocotylus atlanticus	A	*Anas platyrhynchos*	Russia	MH818008	–	[26]
Notocotylus attenuatus	A	*Aythya ferina*	Ukraine	AF184259	–	[12]
Notocotylus cygni	A	*Cygnus melancoryphus*	Argentina	OR501502	–	[27]
Notocotylus ephemera	C	*Planorbis planorbis*	Russia	OP720893	–	[28]
Notocotylus ikutai	C	*Radix auricularia*	Japan	LC596925	–	[29]
Notocotylus intestinalis	C	*Parafossarulus striatulus*	Vietnam	JQ890559	–	[30]
Notocotylus magniovatus	A	*Gallus gallus* dom.	Russia	MH750016	–	[25]
Notocotylus malhamensis	A	*Myodes glareolus*	UK	JQ766939	–	[31]
Notocotylus sp. AK-2017	C	*Ampullaceana balthica*	Norway	KY513158	–	[32]
***Notocotylus* sp.**	**C**	***Ampullaceana balthica***	**Germany**	**PX641062**	–	**This study**
***Notocotylus* sp.**	**C**	***Ampullaceana balthica***	**Germany**	**PX641090**	–	**This study**
***Notocotylus* sp.**	**C**	***Stagnicola palustris***	**Germany**	**PX641066**	–	**This study**
***Notocotylus* sp.**	**C**	***Stagnicola palustris***	**Germany**	**PX641067**	**PX637225**	**This study**
***Notocotylus* sp.**	**C**	***Stagnicola palustris***	**Germany**	**PX641069**	–	**This study**
Notocotylus sp. A MN-2021	R	*Radix auricularia*	Japan	LC599518	–	[33]
Notocotylus sp. BH-2008	C	*Physa gyrina*	USA	EU712725	–	[34]
Notocotylus sp. BU425	R	*Radix auricularia*	Poland	OQ672264	–	[35]
Notocotylus sp. NR1n	R	*Planorbarius corneus*	Russia	OP981949	–	[36]
Notocotylus sp. NR4	R	*Planorbarius corneus*	Russia	OP981952	–	[36]
Notocotylidae gen. sp. OK-2019	C	*Bithynia tentaculata*	Germany	MN726958	–	[8]
Quinqueserialis quinqueserialis	A	*Microtus pennsylvanicus*	Canada	MW934288	–	[37]
**Opisthotrematidae**						
Opisthotrema dujonis	A	*Dugong dugon*	Australia	AY222223		[2]
**Family Plagiorchiidae**						
Lecithopyge rastellus	A	*Bombina variegata*	Ukraine	AF151932	–	[38]

Lecithopyge sp. BO-2024	C	*Ampullaceana balthica*	Germany	PV248741	PV605649	[4]
***Lecithopyge* sp.**	**C**	***Ampullaceana balthica***	**Germany**	**PX641053**	**PX637217**	**This study**
***Lecithopyge* sp.**	**C**	***Ampullaceana balthica***	**Germany**	**PX641071**	**PX637229**	**This study**
***Lecithopyge* sp.**	**C**	***Ampullaceana balthica***	**Germany**	**PX641074**	**PX637230**	**This study**
Haplometra cylindracea	A	*Rana arvalis*	Ukraine	AF151933	–	[38]
Plagiorchis elegans	C	*Lymnaea stagnalis*	Slovakia	KJ533392	–	[39]
Plagiorchis elegans	C	*Lymnaea stagnalis*	Czech Republic	–	KJ533408	[39]
Plagiorchis elegans	C	*Stagnicola fuscus*	Ireland	MW528601	–	[40]
Plagiorchis elegans	**C**	***Lymnaea stagnalis***	**Germany**	**PX641033**	**PX637202**	**This study**
Plagiorchis elegans	**C**	***Lymnaea stagnalis***	**Germany**	**PX641036**	**PX637204**	**This study**
Plagiorchis elegans	**C**	***Stagnicola palustris***	**Germany**	**PX641052**	**PX637216**	**This study**
Plagiorchis elegans	**C**	***Lymnaea stagnalis***	**Germany**	**PX641081**	–	**This study**
Plagiorchis koreanus	C	*Radix auricularia*	Czech Republic	KJ533394	–	[39]
Plagiorchis koreanus	A	*Nyctalus noctula*	Ukraine	AF151930	–	[38]
Plagiorchis koreanus	A	*Myotis daubentonii*	Russia	–	OR515274	[41]
Plagiorchis koreanus	A	*Procyon lotor*	Iran	–	MW390810	[42]
Plagiorchis koreanus	**C**	***Ampullaceana balthica***	**Germany**	**PX641040**	**PX637206**	**This study**
Plagiorchis koreanus	**C**	***Ampullaceana balthica***	**Germany**	**PX641042**	**PX637208**	**This study**
Plagiorchis maculosus	C	*Lymnaea stagnalis*	Czech Republic	KJ533395	–	[39]
Plagiorchis maculosus	C	‘snail’	China	PP060441	–	[43]
Plagiorchis muelleri	A	*Eptesicus serotinus*	Ukraine	AF184250	–	[12]
Plagiorchis muelleri	C	*Ampullaceana balthica*	Ireland	MW528603	–	[40]
Plagiorchis muelleri	C	*Radix auricularia*	Czech Republic	–	PP396768	[44]
Plagiorchis muelleri	A	*Eptesicus nilssoni*	Russia	–	OR515272	[41]
Plagiorchis muelleri	**C**	***Ampullaceana balthica***	**Germany**	**PX641061**	**PX637221**	**This study**
Plagiorchis neomydis	C	*Lymnaea stagnalis*	Slovakia	KJ533397	–	[39]
Plagiorchis vespertilionis	A	*Myotis daubentoni*	Ukraine	AF151931	–	[38]
Plagiorchis vespertilionis	C	*Ampullaceana balthica*	Czech Republic	PP391294	PP396795	[44]
Plagiorchis vespertilionis	C	*Ampullaceana lagotis*	Czech Republic	–	PP396786	[44]

Plagiorchis vespertilionis	**C**	***Ampullaceana balthica***	**Germany**	**PX641068**	**PX637226**	**This study**
Plagiorchis sp. 1 AK-2017	S	*Ampullaceana balthica*	Norway	–	KY513238	[32]
Plagiorchis sp. 2 AK-2017	C	*Ampullaceana balthica*	Norway	KY513164	KY513251	[32]
Plagiorchis sp. 2 OK-2021	C	*Ampullaceana balthica*	Iceland	MW528605	MW520071	[40]
***Plagiorchis* sp. 2**	**C**	***Ampullaceana balthica***	**Germany**	**PX641049**	**PX637213**	**This study**
***Plagiorchis* sp. 2**	**C**	***Ampullaceana balthica***	**Germany**	**PX641065**	**PX637224**	**This study**
***Plagiorchis* sp. 2**	**C**	***Ampullaceana balthica***	**Germany**	**PX641051**	**PX637215**	**This study**
Plagiorchis sp. 3 AK-2017	C	*Ampullaceana balthica*	Norway	KY513166	KY513257	[32]
Plagiorchis sp. 3 OK-2021	C	*Ampullaceana balthica*	Ireland	MW528606	–	[40]
Plagiorchis sp. 3 OK-2021	C	*Ampullaceana balthica*	Iceland	–	MW520079	[40]
***Plagiorchis* sp. 3**	**C**	***Ampullaceana balthica***	**Germany**	**PX641041**	**PX637207**	**This study**
***Plagiorchis* sp. 3**	**C**	***Ampullaceana balthica***	**Germany**	**PX641043**	**PX637209**	**This study**
***Plagiorchis* sp. 3**	**C**	***Ampullaceana balthica***	**Germany**	**PX641044**	**PX637210**	**This study**
Plagiorchis sp. 4 AK-2017	C	*Ampullaceana balthica*	Norway	KY513169	KY513259	[32]
Plagiorchis sp. 5 AK-2017	MC	*Oreodytes alpinus*	Norway	KY513170	–	[32]
Plagiorchis sp. 5 AK-2017	C	*Ampullaceana balthica*	Norway	–	KY513261	[32]
Plagiorchis sp. 5 AK-2017	C	*Ampullaceana balthica*	Norway	–	KY513262	[32]
Plagiorchis sp. 5 OK-2021	C	*Ampullaceana balthica*	Finland	MW528611	–	[40]
Plagiorchis sp. 7 AK-2017	C	*Ampullaceana balthica*	Norway	KY513174	KY513264	[32]
Plagiorchis sp. 7 OK-2021	C	*Ampullaceana balthica*	Ireland	MW528616	MW520083	[40]
***Plagiorchis* sp. 7**	**C**	***Ampullaceana balthica***	**Germany**	**PX641083**	**PX637237**	**This study**
***Plagiorchis* sp. 7**	**C**	***Ampullaceana balthica***	**Germany**	**PX641089**	**PX637240**	**This study**
***Plagiorchis* sp. 7**	**C**	***Ampullaceana balthica***	**Germany**	**PX641050**	**PX637214**	**This study**
Plagiorchis sp. 8 OK-2021	C	*Ampullaceana balthica*	Ireland	MW528619	–	[40]
Plagiorchis sp. 9 OK-2021	C	*Stagnicola fuscus*	Ireland	MW528621	–	[40]
**Pleurogenidae**						
Collyricloides massanae	A	*Erithacus rubecula*	Czech Republic	KP682451	–	[45]
Cortrema magnicaudate	A	*Hirundo rustica*	Czech Republic	KJ700420	–	[46]
Lecithodollfusia arenula	A	*Fulica atra*	Russia	OR233628	–	[47]

Leyogonimus polyoon	A	*Fulica atra*	Poland	KY752116	–	[48]
Leyogonimus polyoon	**C**	***Bithynia tentaculata***	**Germany**	**PX641034**	**PX637203**	**This study**
Leyogonimus polyoon	**C**	***Bithynia tentaculata***	**Germany**	**PX641031**	**PX637201**	**This study**
Leyogonimus polyoon	**C**	***Bithynia tentaculata***	**Germany**	**PX641030**	**PX637200**	**This study**
Macyella postgonoporus	A	*Dendrocopus major*	Czech Republic	KY752115	–	[48]
Parabascus duboisi	A	*Myotis daubentoni*	Ukraine	AY220618	–	[24]
Parabascus duboisi	C	*Bithynia tentaculata*	Germany	MN726967	–	[8]
Parabascus joannae	A	*Myotis daubentoni*	Ukraine	AY220619	–	[24]
Parabascus semisquamosus	A	*Pipistrellus kuhli*	Ukraine	AF151923	–	[38]
Pleurogenidae gen. sp. 1 OK-2019	C	*Bithynia tentaculata*	Germany	MN726968	–	[8]
Pleurogenidae gen. sp. 2 OK-2019	C	*Bithynia tentaculata*	Germany	MN726970	–	[8]
**Prosthogonimidae**						
Prosthohonimus cuneatus	A	*Sturnus vulgaris*	Ukraine	AY220634	–	[24]
Prosthogonimus ovatus	A	*Pica pica*	Ukraine	AF151928	–	[38]
Prosthogonimus ovatus	C	*Bithynia tentaculata*	Germany	MN726975	–	[8]
Prosthogonimus ovatus	C	*Bithynia tentaculata*	Germany	MN726974	–	[8]
Prosthogonimus ovatus	**C**	***Bithynia tentaculata***	**Germany**	**PX641088**	**PX637239**	**This study**
Schistogonimus rarus	A	*Anas querquedula*	Ukraine	AY116869	–	[24]
**Family Psilostomidae**						
Apopharynx bolodes	A	*Fulica atra*	Belarus	PP848221	–	[49]
Neopsilotrema affinis	A	*Aythya affinis*	USA	MW962222	–	[50]
Neopsilotrema lakotae	A	*Aythya americana*	USA	MW962228	–	[50]
Neopsilotrema lisitynae	A	*Anas crecca*	USA	MW962231	–	[50]
Psilochasmus oxyurus	A	*Anas platyrhynchos*	Ukraine	AF151940	–	[38]
Psilostomum brevicolle	A	*Haematopus ostralegus*	Ukraine	KT956950	–	[7]
Sphaeridiotrema aziaticus	A	*Gallus gallus* dom.	Russia	MT986043	–	[51]
Sphaeridiotrema monorchis	A	*Bithynia fuchsiana*	Vietnam	JQ890544	–	[30]
Sphaeridiotrema pseudoglobulus	A	*Aythya affinis*	USA	KT956957	–	[7]
Sphaeridiotrema pseudoglobulus	A	*Aythya affinis*	USA	MW962240	–	[50]

Sphaeridiotrema pseudoglobulus	**C**	***Bithynia tentaculata***	**Germany**	**PX641076**	**PX637232**	**This study**
Sphaeridiotrema pseudoglobulus	**C**	***Bithynia tentaculata***	**Germany**	**PX641087**	**PX637238**	**This study**
Sphaeridiotrema pyriforme	A	*Gallus gallus* dom.	Russia	MT986045	–	[51]
Sphaeridiotrema ussuriense	A	*Gallus gallus* dom.	Russia	MT986039	–	[51]
Sphaeridiotrema sp. OK-2019	C	*Bithynia tentaculata*	Germany	MN726949	–	[8]
Sphaeridiotrema sp. VVT-2015	C	*Bithynia tentaculata*	Lithuania	KT956958	–	[7]
Sphaeridiotrema sp. isolate BV8	MC	*Bithynia tentaculata*	Ireland	PP849707	–	[22]
***Sphaeridiotrema* sp.**	**C**	***Bithynia tentaculata***	**Germany**	**PX641084**	–	**This study**
***Sphaeridiotrema* sp.**	**C**	***Bithynia tentaculata***	**Germany**	**PX641085**	–	**This study**
***Sphaeridiotrema* sp.**	**C**	***Bithynia tentaculata***	**Germany**	**PX641086**	–	**This study**
**Family Rhabdiopoeidae**						
Taprobanella bicaudata	A	*Dugong dugon*	Australia	AY222217	–	[2]
**Family Schistosomatidae**						
Bilharziella polonica	C	*Planorbarius corneus*	England	–	ON987334	[52]
Trichobilharzia australis	A	*Anas superciliosa*	Australia	OK104141	OK357975	[53]
Trichobilharzia franki	A	*Anas platyrhynchos*	Hungary	MZ560941	–	[54]
Trichobilharzia franki	C	*Radix auricularia*	Iran	–	OP740607	[55]
Trichobilharzia franki	A	*Anas platyrhynchos*	Iran	–	MF945593	[56]
Trichobilharzia longicauda	A	*Aythya novaeseelandiae*	New Zealand	OK104148	OK357976	[53]
Trichobilharzia longicauda	A	*Aythya novaeseelandiae*	New Zealand	–	OK357980	[53]
Trichobilharzia novaseelandiae	A	*Aythya novaeseelandiae*	New Zealand	OK104144	OK357972	[53]
Trichobilharzia novaseelandiae	C	*Austropeplea tomentosa*	New Zealand	–	OK357974	[53]
Trichobilharzia ocellata	C	*Lymnaea stagnalis*	Germany	AY157243	AY157189	[57]
Trichobilharzia physellae	A	*Bucephala albeola*	USA	FJ174474	–	[58]
Trichobilharzia physellae	C	*Physella acuta*	Austria	–	OL434665	[59]
Trichobilharzia querquedulae	A	*Spatula cyanoptera*	Chile	PP333195	–	[60]
Trichobilharzia querquedulae	A	*Spatula clypeata*	Iran	–	PP126500	[61]
Trichobilharzia querquedulae	C	*Physa* sp.	Argentina	–	PP333621	[60]
Trichobilharzia regenti	C	*Radix peregra*	Czech Republic	AY157244	–	[57]

Trichobilharzia regenti	–	*Anas clypeata*	Iran	–	MN337552	[62]
Trichobilharzia stagnicolae	A	*Mergus merganser*	USA	FJ174478	–	[58]
Trichobilharzia szidati	C	*Lymnaea stagnalis*	Czech Republic	AY157245	–	[57]
Trichobilharzia szidati	C	*Lymnaea stagnalis*	Russia	–	JF838198	[63]
Trichobilharzia szidati	C	*Lymnaea stagnalis*	Belarus	–	MT708487	[64]
Trichobilharzia sp. A SVB-2009	A	*Anas americana*	USA	FJ174472	–	[58]
Trichobilharzia sp. D SVB-2009	C	*Stagnicola* sp.	Canada	FJ174481	–	[58]
Trichobilharzia sp. E SVB-2009	C	*Stagnicola* sp.	Canada	FJ174480	–	[58]
Trichobilharzia sp.	A	*Netta peposaca*	Argentina	PP333197	PP333623	[60]
Trichobilharzia sp. HAP_2013	C	*Physa marmorata*	Brazil	KJ855994	–	[65]
Trichobilharzia sp. L1	C	*Pseudosuccinea columella*	USA	KY319361	–	[66]
Trichobilharzia sp. L2	C	*Pseudosuccinea columella*	USA	KY319362	–	[66]
Trichobilharzia sp. TB3	C	*Ampullaceana balthica*	England	–	ON987329	[52]
***Trichobilharzia* sp.**	**C**	***Ampullaceana balthica***	**Germany**	**PX641039**	**PX637205**	**This study**
***Trichobilharzia* sp.**	**C**	***Ampullaceana balthica***	**Germany**	**PX641045**	**PX637211**	**This study**
***Trichobilharzia* sp.**	**C**	***Ampullaceana balthica***	**Germany**	**PX641059**	**PX637220**	**This study**
**Family Strigeidae**						
Apatemon gracilis	C	*Ampullaceana balthica*	Norway	KY513175	–	[32]
Apatemon gracilis	**C**	***Ampullaceana balthica***	**Germany**	**PX641029**	**PX637199**	**This study**
Apatemon gracilis	**C**	***Ampullaceana balthica***	**Germany**	**PX641032**	–	**This study**
Apatemon gracilis	**C**	***Ampullaceana balthica***	**Germany**	**PX641048**	**PX637212**	**This study**
Apatemon sp. AK-2017	MC	*Gastrosteus aculeatus*	Norway	KY513178	–	[32]
Apatemon sp. 'jamiesoni'	A	*Phalacrocorax punctatus*	New Zealand	KT334169	–	[67]
Apharyngostrigea cornu	A	*Ardea cinerea*	Ukraine	AF184264	–	[12]
Australapatemon burti	A	*Anas diazi*	Mexico	MF398342	–	[68]
Australapatemon burti	C	*Helisoma trivolvis*	Canada	KY207625	–	[69]
Australapatemon burti	**C**	***Ampullaceana balthica***	**Germany**	**PX641070**	**PX637227**	**This study**
Australapatemon burti	**C**	***Ampullaceana balthica***	**Germany**	**PX641075**	**PX637231**	**This study**
Australapatemon burti	**C**	***Ampullaceana balthica***	**Germany**	**PX641079**	–	**This study**

Australapatemon burti	**C**	***Planorbis carinatus***	**Germany**	–	**PX637228**	**This study**
Cotylurus cornutus	A	*Anas platyrhynchos*	Poland	MW244637	–	[70]
Cotylurus cornutus	A	*Anas platyrhynchos*	Poland	OM949854	–	[71]
Cotylurus cornutus	MC	*Ampullaceana balthica*	Norway	KY513180	–	[32]
Cotylurus hebraicus	A	*Fulica atra*	Poland	MW244639	–	[70]
Cotylurus strigeoides	A	*Anas platyrhynchos*	Poland	MW244640	–	[70]
Cotylurus strigeoides	A	*Anas platyrhynchos*	Poland	OM949853	–	[71]
Cotylurus sp. (lineage II)	MC	*Radix auricularia*	Poland	OM949869	–	[71]
***Cotylurus* sp.**	**C**	***Ampullaceana balthica***	**Germany**	**PX641064**	**PX637223**	**This study**
***Cotylurus* sp.**	**C**	***Ampullaceana balthica***	**Germany**	**PX641063**	**PX637222**	**This study**
***Cotylurus* sp.**	**MC**	***Ampullaceana balthica***	**Germany**	**PX641057**	**PX637219**	**This study**
***Cotylurus* sp.**	**MC**	***Ampullaceana balthica***	**Germany**	**PX641058**	–	**This study**
Parastrigea cincta	A	*Eudocimus albus*	Mexico	MF398347	–	[68]
Parastrigea diovadena	A	*Eudocimus albus*	Mexico	MF398348	–	[68]
Parastrigea plataleae	A	*Platalea ajaja*	Mexico	MF398346	–	[68]
Strigea magnirostris	A	*Rupornis magnirostris*	Mexico	OQ647922	–	[72]
**Family Telorchiidae**						
Opisthiogylphe ranae	A	*Rana arvalis*	Ukraine	AF151929	–	[38]
Opisthiogylphe ranae	MC	*Pelophylax ridibundus*	Russia	MK585340	–	[28]
Opisthiogylphe ranae	C	*Ampullaceana balthica*	Germany	PV248753	–	[4]
Opisthiogylphe ranae	**C**	***Stagnicola palustris***	**Germany**	**PX641055**	–	**This study**
Telorchis assula	A	*Natrix natrix*	Ukraine	AF151915	–	[73]
Telorchis bonnerensis	A	*Chelydra serpentine*	USA	JF820592	–	[74]
Telorchis corti	–	*Radix coreana*	South Korea	ON792561	–	[75]

*nad1 sequences

Supplementary Table S5. Co-infections with two trematode species observed in this study.

Host species	Location name	Trematode species 1	Trematode species 2
Ampullaceana balthica	BOYuhHa	*Cotylurus* sp.	*Notocotylus* sp.
Ampullaceana balthica	BRAohBo	*Lecithopyge* sp.	*Notocotylus* sp. AK-2017
Ampullaceana balthica	BRAohBo	*Lecithopyge* sp.	*Notocotylus* sp.
Ampullaceana balthica	BRAohBo	*Lecithopyge* sp.	*Notocotylus* sp. AK-2017
Ampullaceana balthica	BRAohBo	*Lecithopyge* sp.	*Notocotylus* sp. AK-2017
Ampullaceana balthica	LIEohBo	*Cotylurus* sp.	*Plagiorchis* sp. 3 AK-2017
Ampullaceana balthica	LIEohBo	*Cotylurus* sp.	*Plagiorchis* sp.
Ampullaceana balthica	LIEohBo	*Australapatemon burti*	*Notocotylus* sp. AK-2017
Ampullaceana balthica	BOYuhSp	*Echinoparyphium recurvatum*	*Notocotylus* sp. AK-2017
Ampullaceana balthica	BOYuhHa	*Apatemon gracilis*	*Plagiorchis* sp.

References

[1] Johnson, P.T.J., Calhoun, D.M., Achatz, T.J., Greiman, S.E., Gestos, A., Keeley, W.H., 2024. Outbreak of parasite-induced limb malformations in a declining amphibian species in Colorado. Int. J. Parasitol. Parasit. Wildl. 24, 100965. https://doi.org/10.1016/j.ijppaw.2024.100965.

[2] Olson, P.D., Cribb, T.H., Tkach, V.V., Bray, R.A., Littlewood, D.T., 2003. Phylogeny and classification of the Digenea (Platyhelminthes: Trematoda). Int. J. Parasitol. 33 (7), 733–755. https://doi.org/10.1016/s0020-7519(03)00049-3.

[3] Pérez-Ponce de León, G., Hernández-Mena, D.I., 2019. Testing the higher-level phylogenetic classification of Digenea (Platyhelminthes, Trematoda) based on nuclear rDNA sequences before entering the age of the 'next-generation' Tree of Life. J. Helminthol. 93 (3), 260–276. https://doi.org/10.1017/S0022149X19000191.

[4] Hüsken, A., Schwelm, J., Rückert, S., Sures, B., 2025. Intermediate insights: tracing trematodes infecting amphibians via their first intermediate snail hosts. Parasites Vectors 18, 285. https://doi.org/10.1186/s13071-025-06920-x.

[5] Tandon, V., Athokpam, V.D., Jyrwa, D.B., Thapa, S., 2013. Direct submission.

[6] Achatz, T.J., Pulis, E.E., Junker, K., Binh, T.T., Snyder, S.D., Tkach, V.V., 2019. Molecular phylogeny of the Cyathocotylidae (Digenea, Diplostomoidea) necessitates systematic changes and reveals a history of host and environment switches. Zool. Scr. 48 (4), 545–556. https://doi.org/10.1111/zsc.12360.

[7] Tkach, V.V., Kudlai, O., Kostadinova, A., 2016. Molecular phylogeny and systematics of the Echinostomatoidea Looss, 1899 (Platyhelminthes: Digenea). Int. J. Parasitol. 46, 171–185.

[8] Schwelm, J., Kudlai, O., Smit, N.J., Selbach, C., Sures, B., 2020. High parasite diversity in a neglected host: larval trematodes of *Bithynia tentaculata* in Central Europe. J. Helminthol. 94, e120. https://doi.org/10.1017/S0022149X19001093.
[9] Le, T.H., Pham, L.T.K., Van Quyen, D., Nguyen, K.T., Doan, H.T.T., Saijuntha, W., Blair, D., 2024. The ribosomal transcription units of five echinostomes and their taxonomic implications for the suborder Echinostomata (Trematoda: Platyhelminthes). Parasitol. Res. 123 (1), 103. https://doi.org/10.1007/s00436-023-08110-z.
[10] Kostadinova, A., Herniou, E.A., Barrett, J., Littlewood, D.T., 2003. Phylogenetic relationships of *Echinostoma* Rudolphi, 1809 (Digenea: Echinostomatidae) and related genera re-assessed via DNA and morphological analyses. Syst. Parasitol. 54 (3), 159–176. https://doi.org/10.1023/a:1022681123340.
[11] Enabulele, E.E., Lawton, S.P., Walker, A.J., Kirk, R.S., 2023. Molecular epidemiological analyses reveal extensive connectivity between *Echinostoma revolutum* (sensu stricto) populations across Eurasia and species richness of zoonotic echinostomatids in England. PLOS ONE 18 (2), e0270672. https://doi.org/10.1371/journal.pone.0270672.
[12] Tkach, V., Pawlowski, J., Mariaux, J., Swiderski, Z., 2001. Molecular phylogeny of the suborder Plagiorchiata and its position in the system of Digenea. In: Littlewood, D.T.J, Bray, R.A. (Eds.), Interrelationships of platyhelminthes. Taylor & Francis, London, 186–193.
[13] Georgieva, S., Blasco-Costa, I., Kostadinova, A., 2017. Molecular characterisation of four echinostomes (Digenea: Echinostomatidae) from birds in New Zealand, with descriptions of *Echinostoma novaezealandense* n. sp. and *Echinoparyphium poulini* n. sp. Syst. Parasitol. 94 (4), 477–497. https://doi.org/10.1007/s11230-017-9712-x.
[14] Pantoja, C., Faltýnková, A., O'Dwyer, K., Jouet, D., Skírnisson, K., Kudlai, O., 2021. Diversity of echinostomes (Digenea: Echinostomatidae) in their snail hosts at high latitudes. Parasite 28 (59). https://doi.org/10.1051/parasite/2021054.
[15] McPhail, B.A., Tomusiak, S., Veinot, H., Dodds, N., Hanington, P.C., 2025. Reclaimed wetlands support rich trematode and host diversity: findings from a four-year survey. Int. J. Parasitol. S0020-7519(25)00140-7. https://doi.org/10.1016/j.ijpara.2025.08.006.
[16] Gordy, M.A., Hanington, P.C., 2019. A fine-scale phylogenetic assessment of digenean trematodes in central Alberta reveals we have yet to uncover their total diversity. Ecol. Evol. 9 (6), 3153–3238. https://doi.org/10.1002/ece3.4939.
[17] Georgieva, S., Faltýnková, A., Brown, R., Blasco-Costa, I., Soldánová, M., Sitko, J., Scholz, T., Kostadinova, A., 2014. *Echinostoma 'revolutum'* (Digenea: Echinostomatidae) species complex revisited: species delimitation based on novel molecular and morphological data gathered in Europe. Parasit. Vectors 7, 520. https://doi.org/10.1186/s13071-014-0520-8.
[18] Mohanta, U.K., Watanabe, T., Anisuzzaman, Ohari, Y., Itagaki, T., 2019. Characterization of *Echinostoma revolutum* and *Echinostoma robustum* from ducks in Bangladesh based on morphology, nuclear ribosomal ITS2 and mitochondrial nad1 sequences. Parasitol. Int. 69, 1–7. https://doi.org/10.1016/j.parint.2018.11.002.
[19] Marcilla, A., Trelis, M., Munoz-Antoli, C., Esteban, G., Toledo, R., 2003. Direct submission.
[20] Amer, S., Maza, F., 2017. Direct submission.
[21] Snyder, S.D., Tkach, V.V., 2001. Phylogenetic and biogeographical relationships among some holarctic frog lung flukes (Digenea: Haematoloechidae). J. Parasitol. 87 (6), 1433–1440. https://doi.org/10.1645/0022-3395(2001)087[1433:PABRAS]2.0.CO;2.
[22] Faltýnková, A., O'Dwyer, K., Pantoja, C., Jouet, D., Skírnisson, K., Kudlai, O., 2024. Trematode species diversity in the faucet snail, *Bithynia tentaculata* at the western edge of its native distribution, in Ireland. J. Helminthol. 98, e52. https://doi.org/10.1017/S0022149X24000397.

[23] Kudlai, O., Stunzenas, V., Tkach, V.V., 2015. The taxonomic identity and phylogenetic relationships of *Cercaria pugnax* and *C. helvetica* XII (Digenea: Lecithodendriidae) based on morphological and molecular data. Folia Parasitol. 62 (1). http://dx.doi.org/10.14411/fp.2015.003.

[24] Tkach, V.V., Littlewood, D.T.J., Olson, P.D., Kinsella, M., Zdzislaw, S., 2003. Molecular phylogenetic analysis of the Microphalloidea Ward, 1901 (Trematoda: Digenea). Syst. Parasitol. 56, 1–15. https://doi.org/10.1023/A:1025546001611.

[25] Izrailskaia, A.V., Besprozvannykh, V.V., Tatonova, Y.V., Nguyen, H.M., Ngo, H.D., 2019. Developmental stages of *Notocotylus magniovatus* Yamaguti, 1934, *Catatropis vietnamensis* n. sp., *Pseudocatatropis dvoryadkini* n. sp., and phylogenetic relationships of Notocotylidae Lühe, 1909. Parasitol. Res. 118, 469–481. https://doi.org/10.1007/s00436-018-6182-2.

[26] Gonchar, A., Jouet, D., Skírnisson, K., Krupenko, D., Galaktionov, K.V., 2019. Transatlantic discovery of *Notocotylus atlanticus* (Digenea: Notocotylidae) based on life cycle data. Parasitol. Res. 118 (5), 1445–1456. https://doi.org/10.1007/s00436-019-06297-8.

[27] Flores, V.R., Hernández-Orts, J.S., Viozzi, G.P., 2023. A new species of *Notocotylus* (Digenea: Notocotylidae) from the black-necked swan *Cygnus melancorhyphus* (Molina) of Argentina. Vet. Parasitol. Reg. Stud. Reports 45, 100925. https://doi.org/10.1016/j.vprsr.2023.100925.

[28] Svinin, A.O., Chikhlyaev, I.V., Bashinskiy, I.W., Osipov, V.V., Neymark, L.A., Ivanov, A.Y., Stoyko, T.G., Chernigova, P.I., Ibrogimova, P.K., Litvinchuk, S.N., Ermakov, O.A., 2023. Diversity of trematodes from the amphibian anomaly P hotspot: Role of planorbid snails. PLOS ONE 18(3): e0281740. https://doi.org/10.1371/journal.pone.0281740.

[29] Sasaki, M., Kobayashi, M., Yoshino, T., Asakawa, M., Nakao, M., 2021. *Notocotylus ikutai* n. sp. (Digenea: Notocotylidae) from lymnaeid snails and anatid birds in Hokkaido, Japan. Parasitol. Int. 83, 102318. https://doi.org/10.1016/j.parint.2021.102318.

[30] Besprozvannykh, V., Ngo, H., Nguyen, H., Hung, N., Rozhkovan, K., Ermolenko, A.V., 2013. Descriptions of digenean parasites from three snail species, *Bithynia fuchsiana* (Morelet), *Parafossarulus striatulus* Benson and *Melanoides tuberculata* Müller, in North Vietnam. Helminthologia 50. 190-204. 10.2478/s11687-013-0131-5.

[31] Boyce, K., Hide, G., Craig, P.S., Harris, P.D., Reynolds, C., Pickles, A., Rogan, M.T., 2012. Identification of a new species of digenean *Notocotylus malhamensis* n. sp. (Digenea: Notocotylidae) from the bank vole (*Myodes glareolus*) and the field vole (*Microtus agrestis*). Parasitology 139 (12), 1630–1639. https://doi.org/10.1017/S0031182012000911.

[32] Soldánová, M., Georgieva, S., Roháčová, J., Knudsen, R., Kuhn, J.A., Henriksen, E.H., Siwertsson, A., Shaw, J.C., Kuris, A.M., Amundsen, P.A., Scholz, T., Lafferty, K.D., Kostadinova, A., 2017. Molecular analyses reveal high species diversity of trematodes in a sub-Arctic lake. Int. J. Parasitol. 47 (6), 327–345. https://doi.org/10.1016/j.ijpara.2016.12.008.

[33] Nakao, M., Sasaki, M., 2021. Trematode diversity in freshwater snails from a stopover point for migratory waterfowls in Hokkaido, Japan: An assessment by molecular phylogenetic and population genetic analyses. Parasitol. Int. 83, 102329. https://doi.org/10.1016/j.parint.2021.102329.

[34] Hanelt, B., 2009. Hyperparasitism by *Paragordius varius* (Nematomorpha: Gordiida) larva of Monostome redia (Trematoda: Digenea). J. Parasitol. 95 (1), 242–243. https://doi.org/10.1645/GE-1683.1.

[35] Kanarek, G., Gabrysiak, J., Pyrka, E., Jezewski, W., Stanicka, A., Cichy, A., Zbikowska, E., Zalesny, G., Hildebrand, J., 2023. Hyperparasitism among larval stages of Digenea in snail hosts: sophisticated life strategy or pure randomness? The scenario of *Cotylurus* sp. Zool. J. Linn. Soc. 200 (4). https://doi.org/10.1093/zoolinnean/zlad102.

[36] Vinogradova, A.A., Ataev, G.L., Isakova, N.P., Prokhorova, E.E., 2022. Direct submission.

[37] Gagnon, D.K., Kasl, E.L., Preisser, W.C., Belden, L.K., Detwiler, J.T., 2021. Morphological and molecular characterization of *Quinqueserialis* (Digenea: Notocotylidae) species diversity in North America. Parasitology 148 (9), 1083–1091. https://doi.org/10.1017/S0031182021000792.

[38] Tkach, V., Pawlowski, J., Mariaux, J., 2000. Phylogenetic analysis of the suborder plagiorchiata (Platyhelminthes, Digenea) based on partial lsrDNA sequences. Int. J. Parasitol. 30 (1), 83–93. https://doi.org/10.1016/s0020-7519(99)00163-0.

[39] Zikmundová, J., Georgieva, S., Faltýnková, A., Soldánová, M., Kostadinova, A., 2014. Species diversity of *Plagiorchis* Lühe, 1899 (Digenea: Plagiorchiidae) in lymnaeid snails from freshwater ecosystems in central Europe revealed by molecules and morphology. Syst. Parasitol. 88 (1), 37–54. https://doi.org/10.1007/s11230-014-9481-8.

[40] Kudlai, O., Pantoja, C., O'Dwyer, K., Jouet, D., Skírnisson, K., Faltýnková, A., 2021. Diversity of *Plagiorchis* (Trematoda: Digenea) in high latitudes: Species composition and snail host spectrum revealed by integrative taxonomy. J. Zool. Syst. Evol. Res. 59, 937-962. https://doi.org/10.1111/jzs.12469.

[41] Kirillova, N.Y., Kirillov, A.A., Shchenkov, S.V., Knyazev, A.E., Vekhnik, V.A., 2024. Morphological and molecular characterization of plagiorchiid trematodes (*Plagiorchis*: Plagiorchiidae, Digenea) from bats with redescription of *Plagiorchis mordovii* Shaldybin, 1958. J. Helminthol. 98, e2. doi:10.1017/S0022149X23000913.

[42] Sharifdini, M., Ashrafi, K., Heddergott, M., 2020. Direct submission.

[43] Li, J.K., Zhang, J., Zhang, H., 2023. Direct submission.

[44] Kundid, P., Pantoja, C., Janovcová, K., Soldánová, M., 2024. Molecular diversity of the genus *Plagiorchis* Luehe, 1899 in snail hosts of Central Europe with evidence of new lineages. Diversity 16 (3), 158. https://doi.org/10.3390/d16030158.

[45] Kanarek, G., Zaleśny, G., Czujkowska, A., Sitko, J., Harris, P.D., 2015. On the systematic position of *Collyricloides massanae* Vaucher, 1969 (Platyhelminthes: Digenea) with notes on distribution of this trematode species. Parasitol. Res. 114, 1495–1501. https://doi.org/10.1007/s00436-015-4333-2.

[46] Kanarek, G., Zaleśny, G., Sitko, J., Tkach, V.V., 2014. Phylogenetic relationships and systematic position of the families Cortrematidae and Phaneropsolidae (Platyhelminthes: Digenea). Folia Parasitol. 61 (6), 523–528.

[47] Vlasenkov, S., Калмыков, A., Sokolov, S., 2023. Phylogenetic position of *Lecithodollfusia* (Trematoda: Microphalloidea) inferred from molecular data on *L. arenula* ex *Fulica atra* (Aves: Rallidae). Invertzool 20, 368-379. https://doi.org/10.15298/invertzool.20.4.02.

[48] Kanarek, G., Zaleśny, G., Sitko, J., Tkach, V.V., 2017. The systematic position and structure of the genus *Leyogonimus* Ginetsinskaya, 1948 (Platyhelminthes: Digenea) with comments on the taxonomy of the superfamily Microphalloidea Ward, 1901. Acta Parasitol. 62 (3), 617–624. https://doi.org/10.1515/ap-2017-0075.

[49] Vlasenkov, S.A., Akimova, L.N., Sokolov, S.G., 2024. New record and phylogenetic assessment of *Apopharynx bolodes* (Braun, 1902)(Digenea: Psilostomidae), a parasite of Eurasian Coot *Fulica atra* Linnaeus, 1758 (Aves: Rallidae). J. Helminthol. 98, e65.

[50] Achatz, T.J., Bennett, D.M., Martens, J.R., Sorensen, R.E., Nelson, R.G., Bates, K.M., Serbina, E.A., Tkach, V.V., 2021. Description and phylogenetic affinities of a new species of *Neopsilotrema* (Digenea: Psilostomidae) from Lesser Scaup, Aythya affinis (Anseriformes: Anatidae). J. Parasitol. 107 (4), 566–574. https://doi.org/10.1645/21-25.

[51] Kalinina, K.A., Tatonova, Y.V., Besprozvannykh, V.V., 2022. New species of *Psilotrema* and *Sphaeridiotrema* (Psilostomidae Odhner, 1913) in the east Asian region: Morphology of developmental stages and genetic data. Parasitol. Int. 88, 102554, https://doi.org/10.1016/j.parint.2022.102554.

[52] Juhász, A., Barlow, S.E.J., Williams, H., Johnson, B., Walsh, N.D., Cunningham, L.C., Jones, S., LaCourse, E.J., Stothard, J.R., 2022. A report of *Bilharziella polonica* cercariae in Knowsley Safari, Prescot, United Kingdom, with notes on other trematodes implicated in human cercarial dermatitis. J. Helminthol. 96, e79. https://doi.org/10.1017/S0022149X22000694.

[53] Davis, N.E., Blair, D., Brant, S.V., 2022. Diversity of *Trichobilharzia* in New Zealand with a new species and a redescription, and their likely contribution to cercarial dermatitis. Parasitology 149 (3), 380–395. https://doi.org/10.1017/S0031182021001943.

[54] Reier, S., Haring, E., Billinger, F., Blatterer, H., Duda, M., Gorofsky, C., Grasser, H.P., Heinisch, W., Hörweg, C., Kruckenhauser, L., Szucsich, N.U., Wanka, A., Sattmann, H., 2020. First confirmed record of *Trichobilharzia franki* Müller & Kimmig, 1994, from *Radix auricularia* (Linnaeus, 1758) for Austria. Parasitol. Res. 119 (12), 4135–4141. https://doi.org/10.1007/s00436-020-06938-3.

[55] Aligolzadeh, A., Yakhchali, M., Ashrafi, K., Sharifdini, M., 2022. Direct submission.

[56] Ashrafi, K., Sharifdini, M., Brant, S.V., Noroosta, A.R., 2017. Direct submission.

[57] Lockyer, A.E., Olson, P.D., Ostergaard, P., Rollinson, D., Johnston, D.A., Attwood, S.W., Southgate, V.R., Horak, P., Snyder, S.D., Le, T.H., Agatsuma, T., McManus, D.P., Carmichael, A.C., Naem, S., Littlewood, D.T., 2003. The phylogeny of the Schistosomatidae based on three genes with emphasis on the interrelationships of *Schistosoma* Weinland, 1858. Parasitology 126 (3), 203–224. https://doi.org/10.1017/s0031182002002792.

[58] Brant, S.V., Loker, E.S., 2009. Molecular systematics of the avian schistosome genus *Trichobilharzia* (Trematoda: Schistosomatidae) in North America. J. Parasitol. 95 (4), 941–963. https://doi.org/10.1645/GE-1870.1.

[59] Helmer, N., Blatterer, H., Hörweg, C., Reier, S., Sattmann, H., Schindelar, J., Szucsich, N.U., Haring, E., 2021. First record of *Trichobilharzia physellae* (Talbot, 1936) in Europe, a possible causative agent of cercarial dermatitis. Pathogens 10 (11), 1473. https://doi.org/10.3390/pathogens10111473.

[60] Oyarzún-Ruiz, P., Thomas, R., Santodomingo, A., Zamorano-Uribe, M., Moroni, M., Moreno, L., Muñoz-Leal, S., Flores, V., Brant, S., 2024. Systematics and life cycles of four avian schistosomatids from Southern Cone of South America. J. Helminthol. 98, e47. https://doi.org/10.1017/S0022149X2400035X.

[61] Ajori, F., Ashrafi, K., Sharifdini, M., Rahmati, B., Brant, S.V., 2024. Direct submission.

[62] Fakhar, M., Karamian, M., Gholami, S., Kialashaki, E., 2019. Direct submission.

[63] Korsunenko, A., Chrisanfova, G., Lopatkin, A., Beer, S.A., Voronin, M., Ryskov, A.P., Semyenova, S.K., 2012. Genetic differentiation of cercariae infrapopulations of the avian schistosome *Trichobilharzia szidati* based on RAPD markers and mitochondrial *cox*1 gene. Parasitol. Res. 110 (2), 833–841. https://doi.org/10.1007/s00436-011-2562-6.

[64] Chrisanfova, G., Semyenova, S., 2020. Direct submission.

[65] Pinto, H.A., Brant, S.V., de Melo, A.L., 2014. *Physa marmorata* (Mollusca: Physidae) as a natural intermediate host of *Trichobilharzia* (Trematoda: Schistosomatidae), a potential causative agent of avian cercarial dermatitis in Brazil. Acta Trop. 138, 38–43. https://doi.org/10.1016/j.actatropica.2014.06.002.

[66] Schultz, J.H., Adema, C.M., 2016. Direct submission.
[67] Blasco-Costa, I., Poulin, R., Presswell, B., 2016. Species of *Apatemon* Szidat, 1928 and *Australapatemon* Sudarikov, 1959 (Trematoda: Strigeidae) from New Zealand: linking and characterising life cycle stages with morphology and molecules. Parasitol. Res. 115 (1), 271–289. https://doi.org/10.1007/s00436-015-4744-0.
[68] Hernández-Mena, D.I., García-Varela, M., Pérez-Ponce de León, G., 2017. Filling the gaps in the classification of the Digenea Carus, 1863: systematic position of the Proterodiplostomidae Dubois, 1936 within the superfamily Diplostomoidea Poirier, 1886, inferred from nuclear and mitochondrial DNA sequences. Syst. Parasitol. 94 (8), 833–848.
[69] Gordy, M.A., Locke, S.A., Rawlings, T.A., Lapierre, A.R., Hanington, P.C., 2017. Molecular and morphological evidence for nine species in North American *Australapatemon* (Sudarikov, 1959): a phylogeny expansion with description of the zygocercous *Australapatemon mclaughlini* n. sp. Parasitol. Res. 116 (8), 2181–2198. https://doi.org/10.1007/s00436-017-5523-x.
[70] Pyrka, E., Kanarek, G., Zaleśny, G., Hildebrand, J., 2021. Leeches as the intermediate host for strigeid trematodes: genetic diversity and taxonomy of the genera *Australapatemon* Sudarikov, 1959 and *Cotylurus* Szidat, 1928. Parasit. Vectors 14, 44. https://doi.org/10.1186/s13071-020-04538-9.
[71] Pyrka, E., Kanarek, G., Gabrysiak, J., Jeżewski, W., Cichy, A., Stanicka, A., Żbikowska, E., Zaleśny, G., Hildebrand, J., 2022. Life history strategies of *Cotylurus* spp. Szidat, 1928 (Trematoda, Strigeidae) in the molecular era - Evolutionary consequences and implications for taxonomy. Int. J. Parasitol. Parasit. Wildl. 18, 201–211. https://doi.org/10.1016/j.ijppaw.2022.06.002.
[72] López-Jiménez, A., González-García, M.T., Andrade-Gómez, L., García-Varela, M., 2023. Phylogenetic analyses based on molecular and morphological data reveal a new species of *Strigea* Abildgaard, 1790 (Digenea: Strigeidae) and taxonomic changes in strigeids infecting Neotropical birds of prey. J. Helminthol. 97, e35. doi:10.1017/S0022149X23000196.
[73] Tkach, V.V., Grabda-Kazubska, B., Pawlowski, J., Swiderski, Z., 1999. Molecular and morphological evidence for close phylogenetic affinities of the genera *Macrodera*, *Leptophallus*, *Metaleptophallus* and *Paralepoderma* (Digenea, Plagiorchiata). Acta Parasitol. 44, 170-179.
[74] Pulis, E., Tkach, V.V., Newman, R., 2011. Helminth parasites of the Wood Frog, *Lithobates sylvaticus*, in prairie pothole wetlands of the Northern Great Plains. Wetlands 31, 675-685. 10.1007/s13157-011-0183-6.
[75] Khrebtova, I.S., Kondakov, A.V., 2022. Direct submission.
[76] Tkach, V., Snyder, S.D., Swiderski, Z., 2001. On the phylogenetic relationships of some members of Macroderoididae and Ochetosomatidae (Digenea, Plagiorchioidea). Acta Parasitol. 46, 267–275.
[77] Snyder, S.D., Tkach, V.V. (2001). Phylogenetic and biogeographical relationships among some holarctic frog lung flukes (Digenea: Haematoloechidae). J. Parasitol. 87 (6), 1433–1440. https://doi.org/10.1645/0022-3395(2001)087[1433:PABRAS]2.0.CO;2.
[78] Littlewood, D.T., Curini-Galletti, M., Herniou, E.A., 2000. The interrelationships of proseriata (Platyhelminthes: seriata) tested with molecules and morphology. Mol. Phylogenet. Evol. 16 (3), 449–466. https://doi.org/10.1006/mpev.2000.0802.
[79] Littlewood, D.T., Rohde, K., Clough, K.A., 1997. Parasite speciation within or between host species? Phylogenetic evidence from site-specific polystome monogeneans. Int. J. Parasitol. 27 (11), 1289–1297. https://doi.org/10.1016/s0020-7519(97)00086-6.
[80] Bowles, J., Blair, D., McManus, D.P., 1995. A molecular phylogeny of the human schistosomes. Mol. Phylogenet. Evol. 4 (2), 103–109. https://doi.org/10.1006/mpev.1995.1011.

[81] Kostadinova, A., Herniou, E.A., Barrett, J., Littlewood, D.T.J., 2003. Phylogenetic relationships of *Echinostoma* Rudolphi, 1809 (Digenea: Echinostomatidae) and related genera re-assessed via DNA and morphological analyses. Syst. Parasitol. 54 (3), 159–176. https://doi.org/10.1023/a:1022681123340.

[82] Benke, M., Brändle, M., Albrecht, C., Wilke, T., 2009. Pleistocene phylogeography and phylogenetic concordance in cold-adapted spring snails (*Bythinella* spp.). Mol. Ecol. 18 (5), 890–903. https://doi.org/10.1111/j.1365-294X.2008.04073.x.

[83] Folmer, O., Black, M., Hoeh, W., Lutz, R., Vrijenhoek, R., 1994. DNA primers for amplification of mitochondrial cytochrome c oxidase subunit I from diverse metazoan invertebrates. Mol. Mar. Biol. Biotechnol. 3 (5), 294–299.
