# Supplementary material for: Land use drives trematode dynamics in a restored stream system
Source: Curr Res Parasitol Vector Borne Dis. 2026 Feb 13;9:100357. doi: 10.1016/j.crpvbd.2026.100357 (PMC12954508; doi:10.1016/j.crpvbd.2026.100357)
Supplement: Multimedia component 4 [file mmc4.pdf]

Supplementary file 4

Supplementary Table S17. Summary of GLMM results for trematode species richness across all snail species.

Family:	nbinom2 (log)
Formula:	TremDiv ~ Site_group + Land_use + Cond. + Temp. + DO + pH + Season + SnailDiv + Method + (1 | Month /Location_name) + (1 | Year/Location_name)

AIC	BIC	logLik	-2*log(L)	df.resid
694.2	755.5	-328.1	656.2	167

	R2m	R2c
delta	0.1892711	0.5123523
lognormal	0.2048131	0.5544241
trigamma	0.1709623	0.4627907

Fixed effects:

	Estimate	Std. Error	z-value	P-value	Sign.
(Intercept)	0.707	0.310	2.279	0.0227	*
Site group:mature	0.133	0.271	0.490	0.6242	
Site group: unimpacted	0.393	0.357	-1.099	0.2716	
Land use: agriculture	0.877	0.364	2.409	0.0160	*
Land use:forest	0.383	0.377	1.016	0.3094	
Cond.	0.086	0.085	1.017	0.3090	
Temp.	0.099	0.086	1.151	0.2497	
DO	0.067	0.067	1.002	0.3163	
pH	0.009	0.054	0.169	0.8661	
Season:autumn	-0.198	0.163	-1.213	0.2252	
Season:summer	-0.093	0.150	-0.622	0.5338	
Season:winter	-0.283	0.176	-1.605	0.1085	
SnailDiv	-0.031	0.044	-0.710	0.4780	
Method: MRR	-0.277	0.149	-1.855	0.0636	.

Random effects:

Groups	Variance	Std. Dev.	Number of. obs.
Location_name:Month	1.455e-07	3.815e-07	95
Month	7.005e-08	2.647e-04	12
Location_name:Year	2.583e-01	5.083e-01	25
Year	4.679e-09	6.841e-05	3

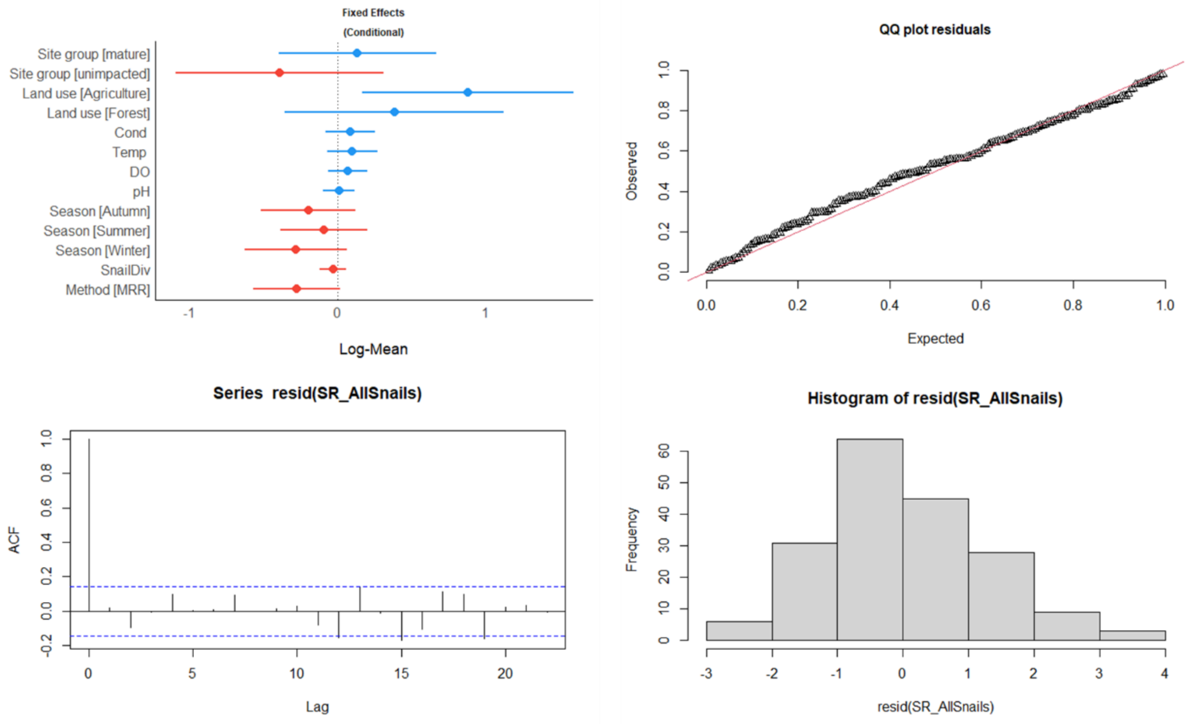

Supplementary Figure S12. Fixed effect estimates and diagnostic plots for the GLMM of trematode species richness across all snail species.

Supplementary Table S18. Summary of GLMM results for trematode overall prevalence across all snail species.

Family:	binomial (logit)				
Formula:	cbind(Infected, TotalSamples - Infected) ~ Site_group + Land_use + Cond. + Temp. + DO + pH + Season + SnailDiv + Method + (1 | Month /Location_name) + (1 | Year/Location_name)				
AIC	BIC	logLik	-2*log(L)	df.resid	
1074.6	1132.7	-519.3	1038.6	168	
	R2m	R2c			
theoretical	0.2514934	0.9603648			
delta	0.2421586	0.9247186			
Fixed effects:					
	Estimate	Std. Error	z-value	P-value	Sign.
(Intercept)	-1.027	0.680	-1.510	0.1313	
Site group:mature	-0.186	0.333	-0.557	0.5776	
Site group: unimpacted	-0.818	0.538	-1.520	0.1285	
Land use: agriculture	1.358	0.565	2.404	0.0162	*
Land use:forest	0.829	0.562	1.474	0.1405	
Cond.	-0.093	0.102	-0.913	0.3610	
Temp.	-0.045	0.126	-0.360	0.7192	
DO	0.182	0.071	2.566	0.0103	*
pH	0.052	0.062	0.847	0.3971	
Season:autumn	-0.690	0.404	-1.711	0.0871	.
Season:summer	-0.220	0.376	-0.583	0.5596	
Season:winter	-0.918	0.396	-2.318	0.0205	*
SnailDiv	-0.147	0.051	-2.918	0.0035	**
Method: MRR	-1.154	0.235	-4.901	9.52e-07	***

Random effects:			
Groups	Variance	Std. Dev.	Number of. obs.
Location_name:Month	0.4096	0.6400	95
Month	0.1187	0.3445	12
Location_name:Year	0.6832	0.8266	25
Year	0.5814	0.7625	3

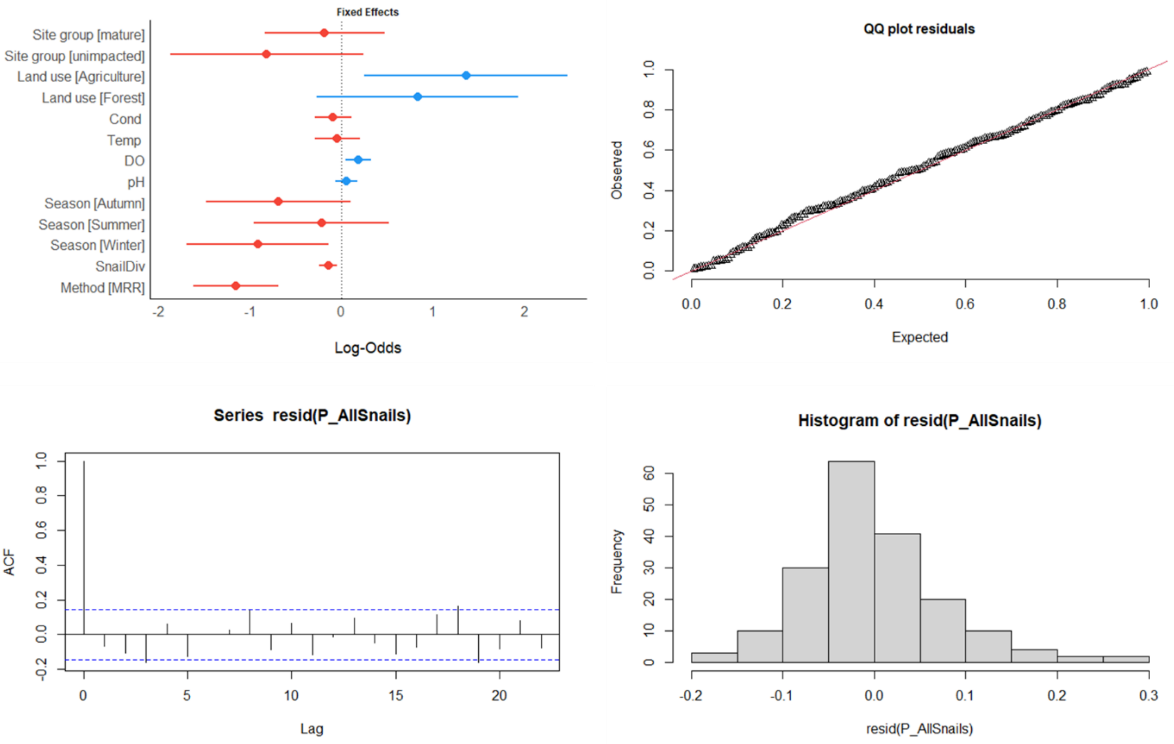

Supplementary Figure S13. Fixed effect estimates and diagnostic plots for the GLMM of trematode prevalence across all snail species.

Supplementary Table S19. Summary of GLMM results for trematode species richness in *Ampullaceana balthica*.

Family:	nbinom2 (log)				
Formula:	TremDiv ~ Site_group + Land_use + Cond. + Temp. + DO + pH + Season + Avg_Host_length + Method + (1 | Month/Location_name) + (1 | Year/Location_name)				
AIC	BIC	logLik	-2*log(L)	df.resid	
633.4	693.1	-297.7	595.4	152	
R2m R2c<br>delta 0.2894682 0.3972919<br>lognormal 0.3144097 0.4315238<br>trigamma 0.2617968 0.3593132					
Fixed effects:					
	Estimate	Std. Error	z-value	P-value	Sign.
(Intercept)	0.722	0.217	3.333	0.0008	***
Site group:mature	0.182	0.207	0.879	0.3809	
Site group:unimpacted	-0.220	0.256	-0.859	0.3901	
Land use:agriculture	0.432	0.245	1.761	0.0782	.

Land use:forest	-0.039	0.281	-0.139	0.8893	
Cond.	0.161	0.083	1.935	0.0529	.
Temp.	0.029	0.090	0.323	0.7470	
DO	0.045	0.069	0.650	0.5157	
pH	0.014	0.058	0.245	0.8065	
Season:autumn	0.070	0.188	0.374	0.7084	
Season:summer	0.105	0.179	0.585	0.5582	
Season:winter	-0.054	0.200	-0.720	0.7868	
Avg_host_length	0.223	0.076	2.936	0.0033	**
Method:MRR	-0.241	0.133	-1.812	0.0700	.
Random effects:					
Groups	Variance	Std. Dev.	Number of. obs.		
Location_name:Month	4.205e-08	2.051e-04	91		
Month	4.445e-07	6.667e-04	12		
Location_name:Year	5.684e-02	2.384e-01	25		
Year	8.100e-14	2.846e-07	3		

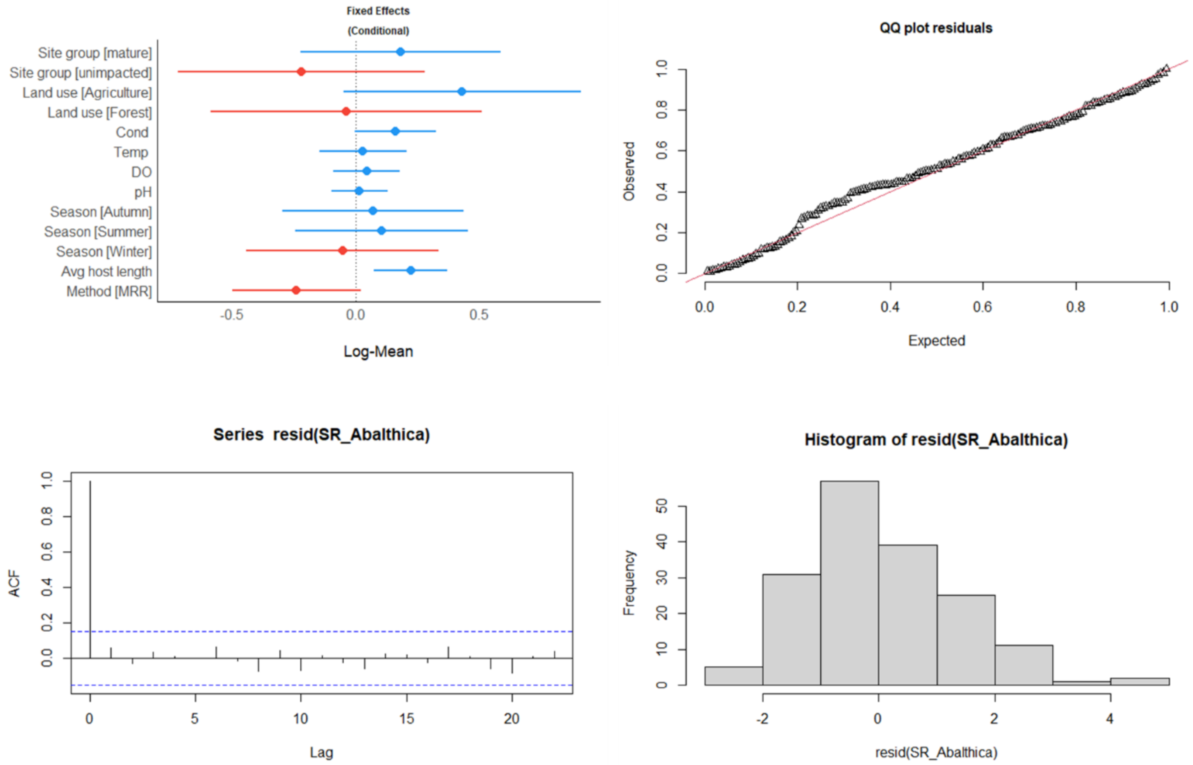

Supplementary Figure S14. Fixed effect estimates and diagnostic plots for the GLMM of trematode species richness in *Ampullaceana balthica*.

Supplementary Table S20. Summary of GLMM results for trematode prevalence in *Ampullaceana balthica*.

Family:	binomial (logit)			
Formula:	cbind(Infected, TotalSamples - Infected) ~ Site_group + Land_use + Cond. + Temp. + DO + pH + Season + Avg_Host_length + Method + (1 | Month/Location_name) + (1 | Year/Location_name)			
AIC	BIC	logLik	-2*log(L)	df.resid
886.6	943.1	-425.3	850.6	153

	R2m	R2c			
theoretical	0.5241705	0.9329722			
delta	0.5053472	0.8994685			
Fixed effects:					
	Estimate	Std. Error	z-value	P-value	Sign.
(Intercept)	-1.493	0.536	-2.786	0.0053	**
Site group:mature	-0.267	0.297	-0.896	0.3701	
Site group:unimpacted	-0.416	0.378	-1.101	0.2709	
Land use:agriculture	1.176	0.381	3.089	0.0020	**
Land use:forest	0.720	0.400	1.797	0.0724	.
Cond.	0.096	0.110	0.875	0.3813	
Temp.	-0.220	0.133	-1.658	0.0974	.
DO	0.048	0.077	0.621	0.5345	
pH	0.029	0.062	0.463	0.6437	
Season:autumn	0.584	0.439	1.331	0.1832	
Season:summer	0.710	0.418	1.699	0.0894	.
Season:winter	-0.323	0.417	-0.776	0.4379	
Avg_host_length	0.765	0.093	8.226	<2e-16	***
Method:MRR	-1.107	0.270	-4.104	4.07e-05	***
Random effects:					
Groups	Variance	Std. Dev.	Number of. obs.		
Location_name:Month	0.2301	0.4797	91		
Month	0.1599	0.3998	12		
Location_name:Year	0.2112	0.4596	25		
Year	0.2646	0.5144	3		

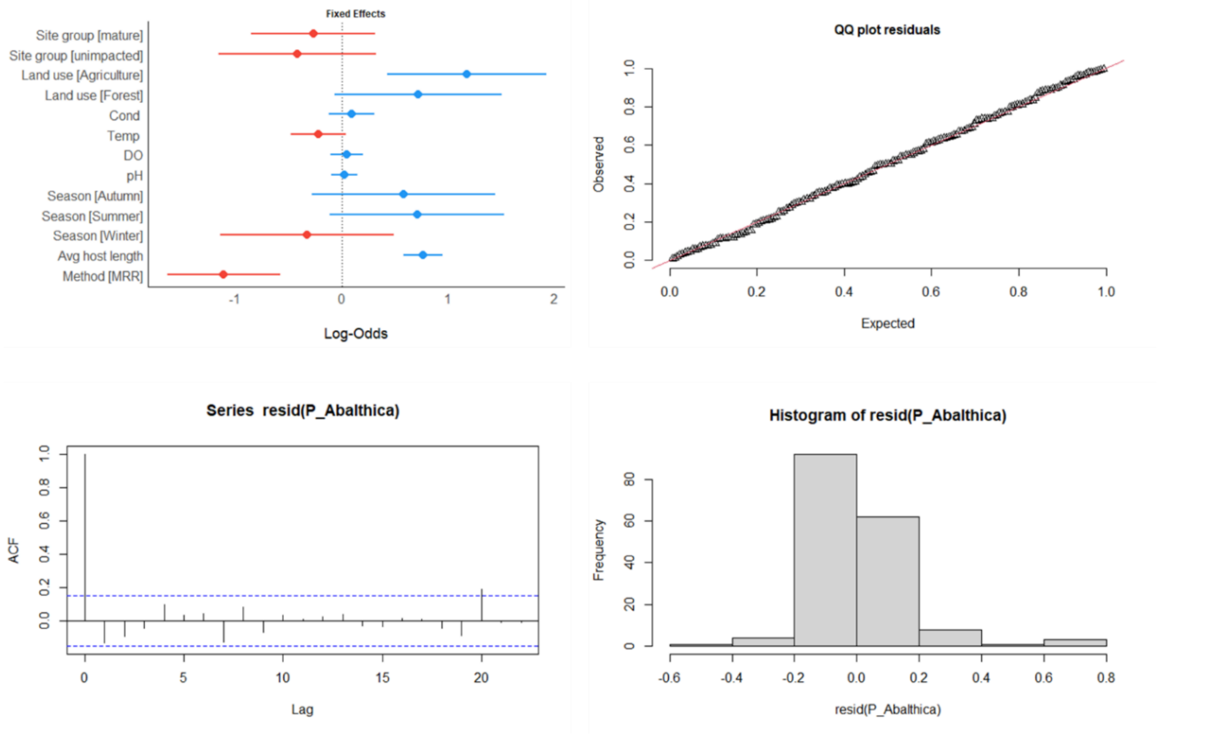

Supplementary Figure S15. Fixed effect estimates and diagnostic plots for the GLMM of trematode species richness in *Ampullaceana balthica*.

Supplementary Table S21. Summary of GLMM results for Shannon diversity across component communities.

Family:	tweedie (log)				
Formula:	Shannon ~ Site_group + Land_use + Cond. + Temp. + DO + pH + Season + Avg_Host_length + Method + Host_species + (1 | Month/Location_name) + (1 | Year/Location_name)				
AIC	BIC	logLik	-2*log(L)	df.resid	
333.8	407.6	-144.9	289.8	193	
	R2m	R2c			
theoretical	NA	NA			
delta	NA	NA			
Fixed effects:					
	Estimate	Std. Error	z-value	P-value	Sign.
(Intercept)	-1.187	1.577	-0.753	0.4517	
Site group:mature	0.175	0.217	0.804	0.4216	
Site group:unimpacted	0.052	0.270	0.191	0.8482	
Land use:agriculture	0.038	0.231	0.163	0.8704	
Land use:forest	-0.403	-1.423	-1.423	0.1548	
Cond.	0.002	0.001	1.837	0.0663	.
Temp.	0.009	0.023	0.409	0.6828	
DO	0.020	0.037	0.546	0.5851	
pH	-0.061	0.165	-0.369	0.7123	
Season:summer	0.131	0.240	0.546	0.5850	
Season:autumn	0.033	0.234	0.139	0.8895	
Season:winter	-0.025	0.275	-0.093	0.9263	
Avg_host_length	0.068	0.036	1.861	0.0627	.
Method:MRR	-0.209	0.166	-1.259	0.2082	
Host_species: B.tentaculata	-0.928	0.469	-1.978	0.0479	*
Host_species: L.stagnalis	-3.875	0.988	-3.920	8.85e-05	***
Host_species: S. palustris	-1.736	0.402	-4.318	1.58e-05	***
Random effects:					
Groups	Variance	Std. Dev.	Number of. obs.		
Location_name:Month	2.796e-14	1.672e-07	86		
Month	7.642e-07	8.742e-04	12		
Location_name:Year	4.649e-27	6.818e-14	24		
Year	4.635e-07	6.808e-04	33		

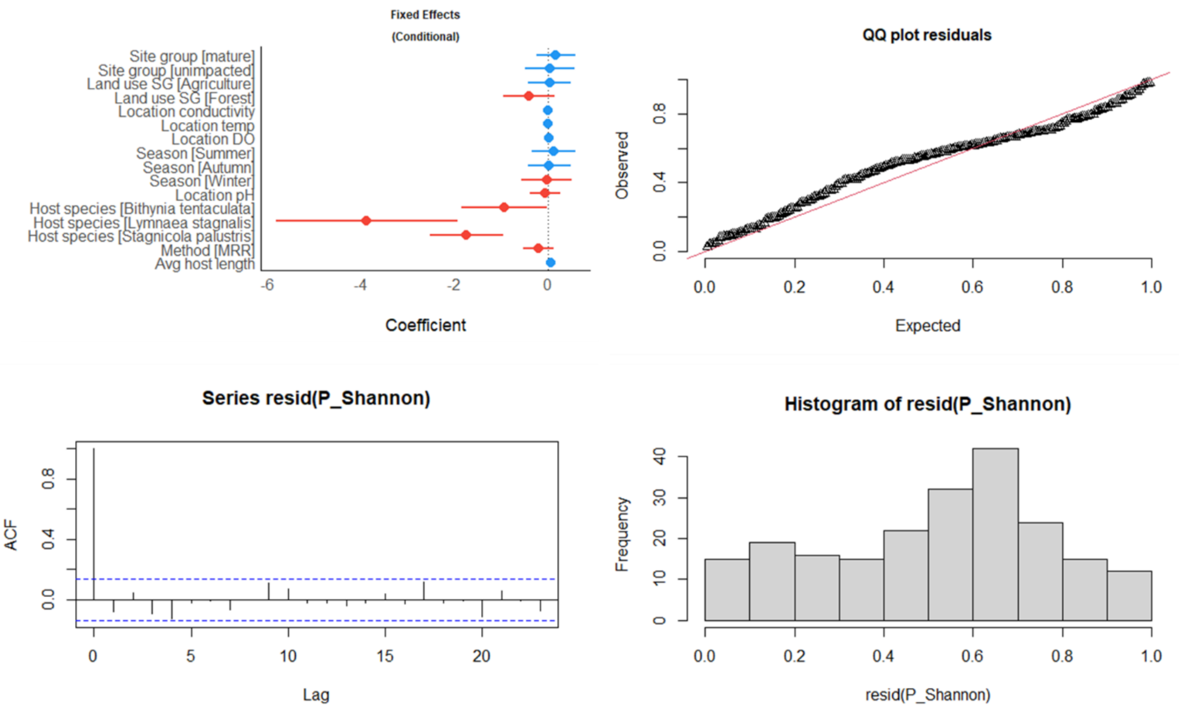

Supplementary Figure S16. Fixed effect estimates and diagnostic plots for the GLMM on Shannon diversity across component communities.

Supplementary Table S22. Summary of GLVMM results for community composition (trematode abundance).

Family:	nbinom2 (log)
Formula:	abundance ~ Site_group + Land_use + Cond. + Temp. + DO + pH + Season + Avg_Host_length + Method + Host_species + (1 | Month/Location_name) + (1 | Year/Location_name)

AIC	BIC	logLik	-2*log(L)	df.resid
4861.2	4994.2	-2410.6	4821.2	5692

	R2m	R2c
delta	0.036658794	0.036659547
lognormal	0.188657845	0.188661722
trigamma	0.002101185	0.002101228

Fixed effects:

	Estimate	Std. Error	z-value	P-value	Sign.
(Intercept)	-5.322	1.402	-3.796	0.0001	
Site group:mature	0.088	0.205	0.432	0.6660	
Site group:unimpacted	0.244	0.240	1.015	0.3099	
Land use:agriculture	0.383	0.205	1.865	0.0621	.
Land use:forest	0.039	0.251	0.156	0.8764	
Cond.	0.004	0.002	1.889	0.0589	.
Temp.	0.029	0.022	1.334	0.1821	
DO	0.037	0.034	1.102	0.2707	
pH	0.083	0.145	0.572	0.5674	
Season:summer	0.211	0.205	1.034	0.3014	
Season:autumn	0.360	0.203	1.773	0.0762	.
Season:winter	0.033	0.235	0.140	0.8887	

Avg_host_length	0.131	0.030	4.370	1.24e-05	***
Method:MRR	-0.220	0.139	-1.580	0.1142	
Host_species: B.tentaculata	-0.826	0.332	-2.491	0.0127	*
Host_species: L.stagnalis	-4.364	0.660	-6.607	3.93e11	***
Host_species: S.palustris	-0.220	0.258	-7.662	1.83e-14	***
Random effects:					
Groups	Variance	Std. Dev.	Number of. obs.		
Location_name:Month	1.008e-06	0.001004	86		
Month	2.710e-02	0.164622	12		
Location_name:Year	1.306e-01	0.361330	24		
Year	1.689e-04	0.012998	33		

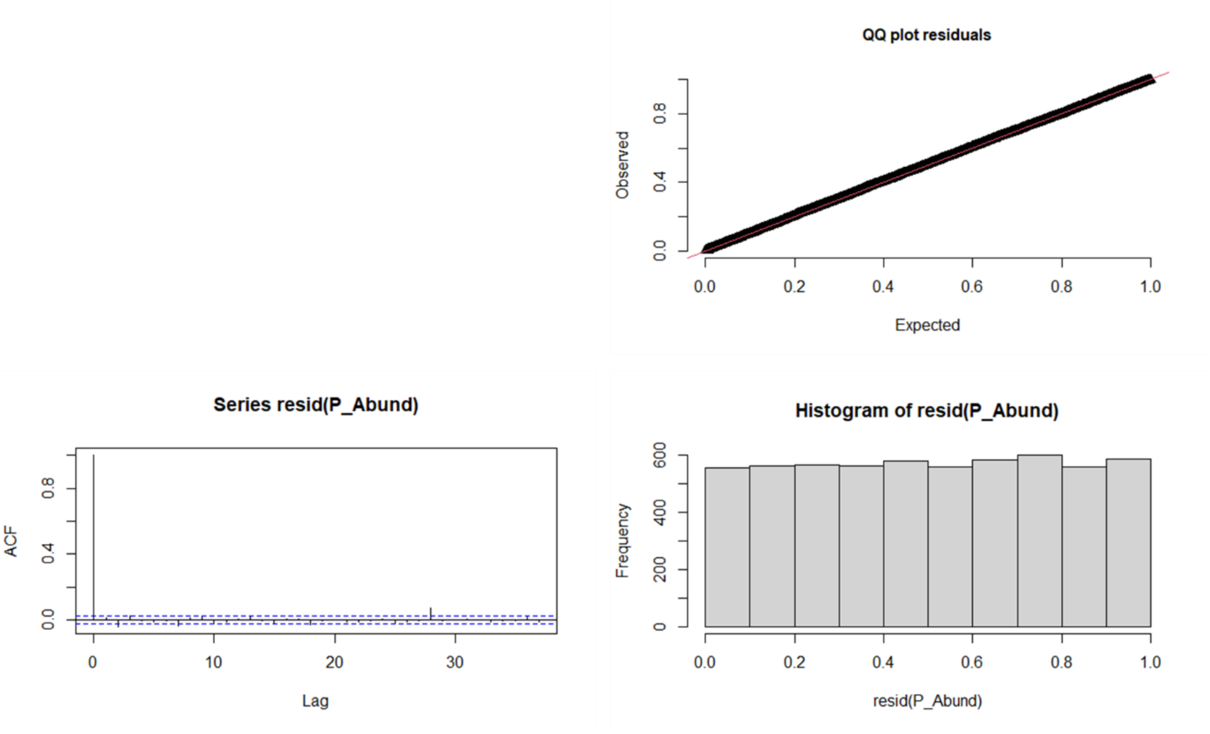

Supplementary Figure S17. Fixed effect estimates and diagnostic plots for the GLVMM on community composition (trematode abundance).
